# DNA Barcoding Provides Taxonomic Clues for Identifying Five Endangered *Phoebe* Species in Southern China

**DOI:** 10.3390/plants14182895

**Published:** 2025-09-18

**Authors:** Wenxiu Yin, Chungui Du, Xiaofeng Zhang, Wenbiao Zhang, Wenwu Wu, Chongrong Fang, Xingcui Xiao, Jiawei Zhu, Fei Yang, Mingzhe Zhang

**Affiliations:** 1 Bamboo Industry Institute, Zhejiang A & F University, Hangzhou 311300, China; 2 Zhejiang Academy of Science and Technology for Inspection and Quarantine, Hangzhou 311202, China; 3 State Key Laboratory of Subtropical Silviculture, College of Forestry and Biotechnology, Zhejiang A & F University, Hangzhou 311300, China; 4 Zhejiang Academy of Forestry, Hangzhou 310023, China; 5 Sichuan Academy of Forestry Sciences, Chengdu 610081, China; 6 Technology Center of Hangzhou Customs, Hangzhou 311202, China

**Keywords:** DNA barcoding, *Phoebe*, species identification, divergent hotspot, phylogeny

## Abstract

Trees in the genus *Phoebe* of the Lauraceae family are commonly known as “Nanmu” in traditional Chinese culture. As they have offered highly valued timbers for construction, furniture, and coffins since the pre-Qin Dynasty, it is crucial to identify and protect these *Phoebe* species. However, the accuracy of *Phoebe* species identification is frequently hampered due to the limitations of traditional morphological and wood anatomy methods as the marker characteristics are very similar between the species, alongside the requirement for specialized expertise. Here, we use DNA barcoding technology for the rapid and accurate identification of five endangered *Phoebe* species in China, including *Phoebe bournei*, *P. chekiangensis*, *P. hui*, *P. sheareri* and *P. zhennan*. Four highly divergent regions (*petA*-*psbJ-psbL-psbF-psbE*, Ψ*ycf1-ndhF*, *rpl32*-*trnL*^UAG^ and *ycf1*) were identified from a comparison of the 20 *Phoebe* plastomes downloaded from the database. Furthermore, phylogenetic analysis on 20 *Phoebe* species showed that *rpl32-trnL^UAG^* + *ycf1*, as well as *rpl32-trnL^UAG^* + *ycf1* + Ψ*ycf1-ndhF*, effectively distinguished the fifteen *Phoebe* species. We further validated the usefulness of the core 2-locus barcode using wood and leaf samples from multiple sites for five target species. The study confirms the reliability of molecular diagnostics for five *Phoebe* species. It also establishes critical taxonomic protocols for conserving these endangered Nanmu species in southern China.

## 1. Introduction

Woody plants are essential to human societies due not only to their ecological functions but also their ornamental and economic value [1]. In China, trees in the genus *Phoebe*, Lauraceae, provide highly valued timbers that have been used nationwide for constructions, furniture, and coffins since the pre-Qin Dynasty (c.a. 221 BC) [2]. The wood of *Phoebe* species, along with *Machilus* in the Lauraceae, are traditionally called “Nanmu” in Chinese culture. Because giant “Nanmu” trees were extensively harvested for palace constructions by imperial families of Ming and Qing Dynasty during the 15th–19th centuries [1,2,3], very few old trees survive in present-day villages and cities [3]. Out of the 34 *Phoebe* species endemic to China*,* four species have been frequently identified as vulnerable species in China’s National Key Protected Wild Plant List and The International Union for Conservation of Nature (IUCN) plant red lists (Supplementary Table S1) [4,5], namely *Phoebe bournei* (Hemsl.) Yen C. Yang, *P. chekiangensis* C. B. Shang, *P. hui* W. C. Cheng ex Yen C. Yang, and *P. zhennan* S. K. Lee & F. N. Wei. Moreover, more than 99% of old *Phoebe* trees (>100-year old) in China are *P. bournei, P. chekiangensis*, *P. sheareri* (Hemsl.) Gamble, and *P. zhennan* [6]. Therefore, it is crucial to accurately identify and protect these tree species.

Distinguishing *Phoebe* species from one to another, however, remains a great challenge, especially for non-taxonomists and non-wood anatomists. Both morphological and anatomical traits overlap significantly in this genus and even across several closely related genera [7,8,9]. For example, *P. bournei* and *P. zhennan* display very similar leaf morphology [2], a relatively stable feature in comparison to flowers and fruits whose observation is constrained by the season [10,11,12,13,14]. In addition, anatomical structures of *Phoebe* wood are even similar to those of *Machilus* (e.g., *P. zhennan* and *M. pingii*, the latter is a synonym of *M. nanmu* (Oliv.) Hemsl.). Because of this, wood anatomy-based techniques have been of little practical use for distinguishing *Phoebe* species [15], hampering the accurate identification of these species for wood trading.

In recent years, studies have attempted to identify the Lauraceae plants using promising DNA techniques due to their highly similar morphological traits. Numerous studies have focused on complete chloroplast genomes from leaves of the Lauraceae plants [7,16,17,18,19,20]. Although these analyses provide informative taxonomic and evolutionary clues about the *Phoebe* species, the sequencing of complete chloroplast genomes is generally time-consuming and labor-intensive [21,22,23,24]. Similar cost and labor issues also occur with the extraction of ancient DNA using hybridization, which is particularly important for identifying archeological “Nanmu” wood [1].

Another set of studies employed DNA barcoding, a method based on the analysis of short DNA sequences [10,25]. DNA barcoding can distinguish closely related species with high accuracy and efficiency [25,26,27,28]. Yet, the main challenge of DNA barcoding arises from the difficulty of finding universal barcodes that can be used to identify all plant species [25]. Efforts have suggested the usefulness of chloroplast DNA markers (e.g., maturase K (*matK*), ribulose-bisphosphate carboxylase (*rbcL*), and the non-coding spacer *psbA-trnH* and nuclear ribosomal DNA marker (e.g., Internal Transcribed Spacer (ITS)), or a combination of these markers for plant classification [12,29,30,31,32]. Consequently, additional markers, known as specific barcodes, are still required to increase the taxonomic resolution [25]. However, current DNA barcoding studies on the *Phoebe* genus have not provided a straightforward approach for distinguishing these five endangered *Phoebe* species [7].

In this study, we investigate the potential of DNA barcoding methodology for distinguishing the wood of the five protected species in the genus *Phoebe*, Lauraceae, including *P. bournei, P. chekiangensis*, *P. hui*, *P. sheareri,* and *P. zhennan,* to complement species identification based on traditional morphology and wood anatomy. In doing so, we first designed specific primers from whole plastomes (widely referred to as complete chloroplast genome sequences [33]) of 20 *Phoebe* species obtained from National Center for Biotechnology Information (NCBI) platform (https://www.ncbi.nlm.nih.gov/, accessed on 4 July 2024), such that the designed barcodes could be highly species-specific. We then tested the capability of the single barcodes and their combinations to identify the five target *Phoebe* species and validated the theoretical barcodes using wood and leaf samples collected from multiple sites in southern China.

## 2. Results

### 2.1. Comparison of Characteristic and Structure of Plastomes

**Figure 1:**
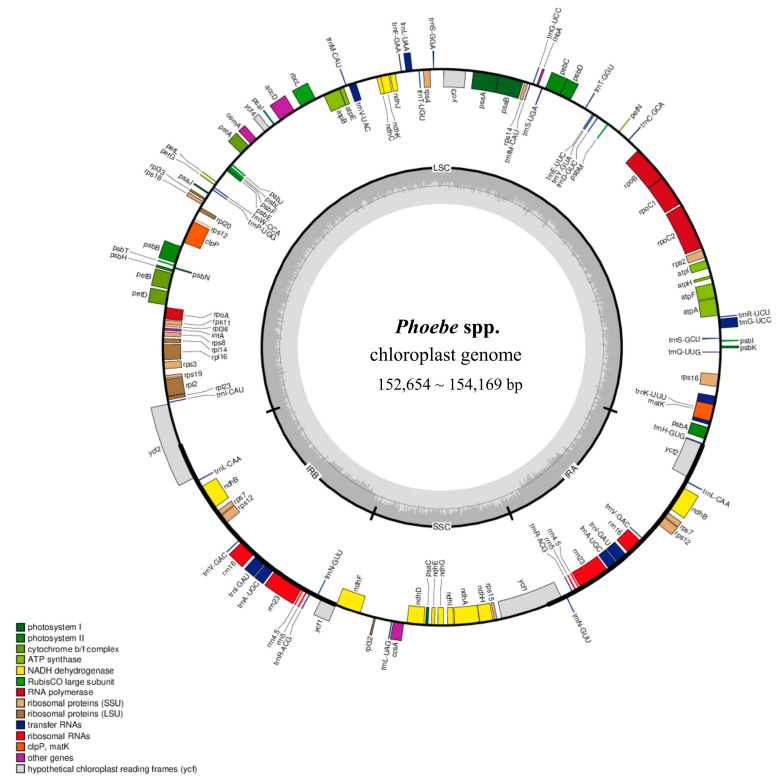
Gene maps based on the chloroplast genomes of 20 *Phoebe* species. The quadripartite structure comprises the large single copy (LSC), small single copy (SSC), and two inverted repeat (IRA and IRB) regions. Genes are shown in different colors at the outer ring according to functional groups, with clockwise-transcribed genes outsides and counterclockwise-transcribed genes insides. GC content is shown in darker gray shade while AT content is lighter at the inner ring.

**Figure 2:**
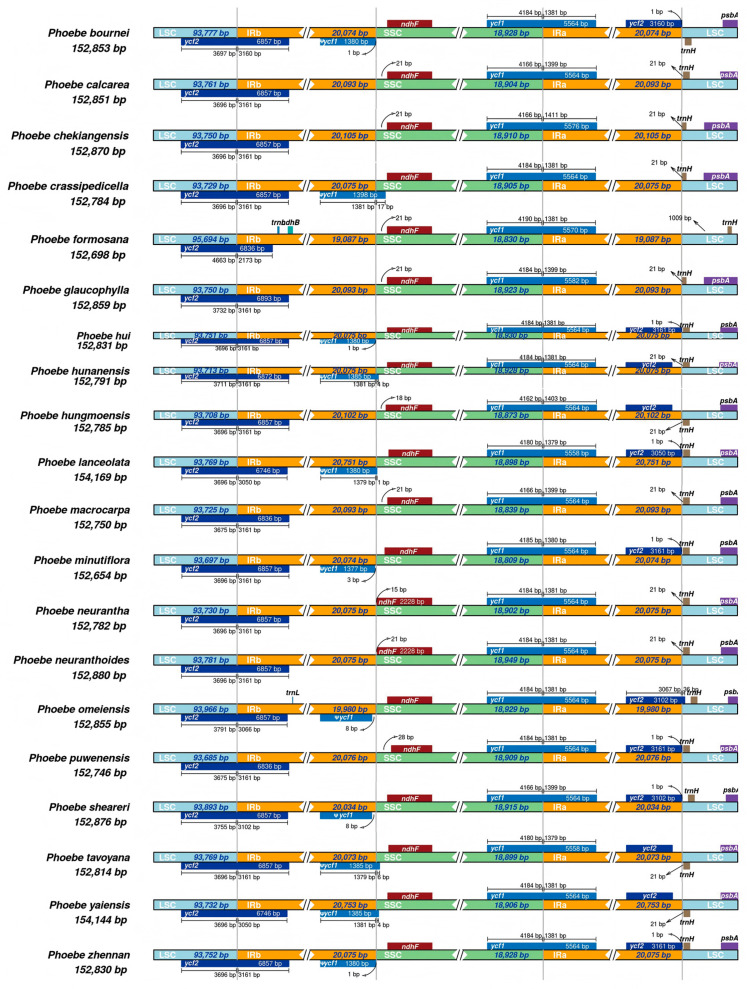
Comparison of the inverted repeat/single copy in the chloroplast genomes of 20 *Phoebe* species. SSC: short single copy; LSC: large single copy.

The 55 plastomes of the 20 *Phoebe* species showed similar sequence characteristics and shared a typical quadripartite structure (Figure 1, Supplementary Figure S1). The sequence lengths range from 152,654 to 154,169 bp and the GC contents range between 39.10 and 39.20%. The gene count within each plastome includes 81 to 84 coding sequences (CDSs), 6 or 8 rRNAs, and 36 tRNAs (Supplementary Table S2). Despite these similarities, significant variations were found near the boundaries between large single-copy (LSC), small single-copy (SSC), and two inverted repeats (IRa and IRb) within the quadripartite structure, thus influencing the lengths of the coding genes *ycf1* and *ycf2* (Figure 2). In addition, the distances from the coding gene *ndhF* to the IR-SSC boundary and *trnH* to the IR-LSC boundary are different among species, ranging from 15 to 21 bp and 21 to 1009 bp, respectively.

### 2.2. Candidate Hotspot Regions

**Figure 3:**
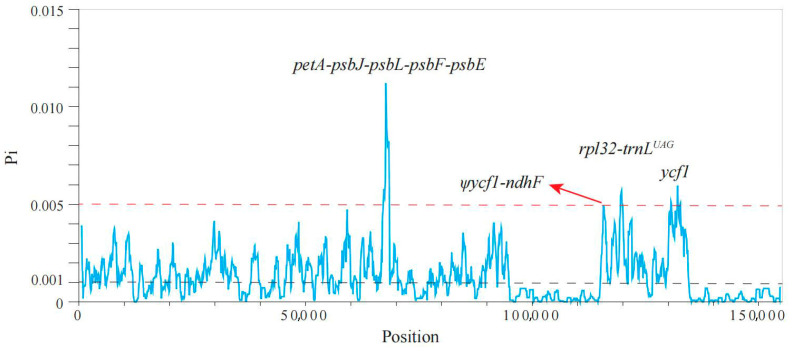
Nucleotide diversity (Pi) of chloroplast genomes in the 20 *Phoebe* species. The black and red dashed lines represent the Pi values of 0.001 and 0.005, respectively.

Despite the similarities among the plastome sequences of the 20 *Phoebe* species (Supplementary Material S2), we identified several candidate hotspot regions showing Pi values greater than 0.005 compared to an averaged Pi value of 0.001 for the whole plastomes. These candidate loci exhibit high variability and may have the potential for species delimitation, including three intergenic regions (*petA*-*psbJ-psbL-psbF-psbE*, Ψ*ycf1*-*ndhF* and *rpl32*-*trnL*^UAG^) and one CDS of *ycf1* (Figure 3).

We further calculated intra- and inter-specific genetic distance for the four candidate loci and their different combinations (Table 1). The barcodes Ψ*ycf1-ndhF*, *rpl32-trnL^UAG^*, and *ycf1* alone show higher average inter-specific than intra-specific distances, while *petA-psbJ-psbL-psbF-psbE* resulted in a higher average intra-specific distance and the lowest identification success rate (ISR = 16.36%). This suggests that candidate loci need to be screened for a Pi value of 0.005 or more in order to identify barcodes capable of species distinction among the 20 *Phoebe* species tested in this study. Although combinations of barcodes do not seem to increase the inter-specific distances substantially, the ISRs are significantly higher, ranging within 49.09–70.91% and 63.64–78.18% for the combinations of two and three loci, respectively. The combination of four loci yielded the same ISR as for the combination of *petA-psbJ-psbL-psbF-psbE* + *rpl32-trnL^UAG^* + *ycf1*. However, we noticed that none of the single-locus barcodes or their combinations exhibited a higher minimum inter-specific distance compared with the maximum intra-specific distance, most likely due to the high degree of genetic similarity in the *Phoebe* genus.

### 2.3. Phylogeny Analysis

**Figure 4:**
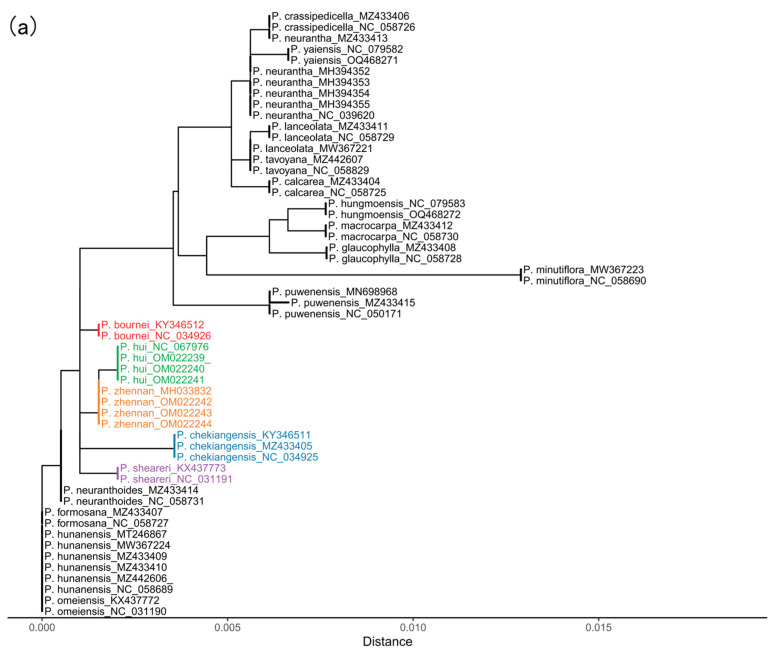

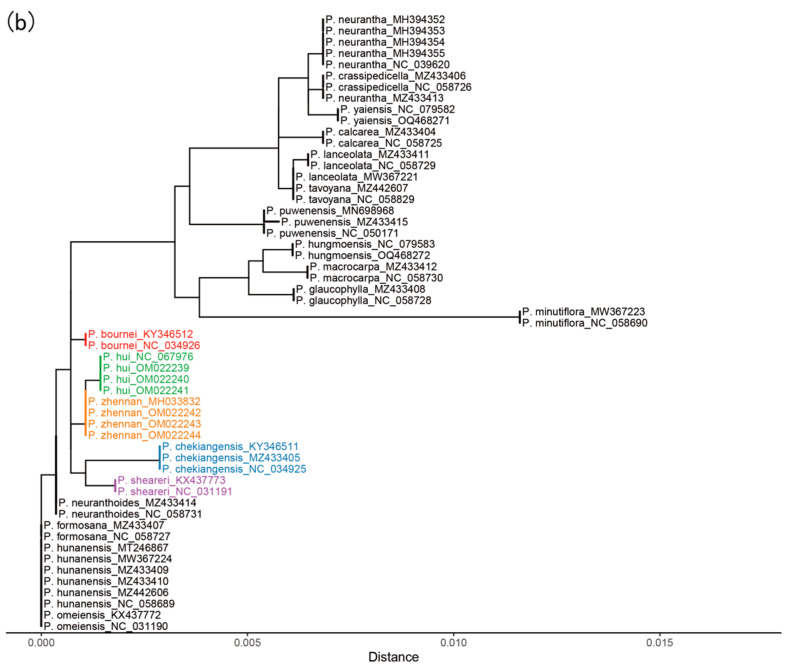
Neighbor-joining (NJ) trees based on multi loci: (**a**) *rpl32-trnL^UAG^* + *ycf1* (**b**) Ψ*ycf1-ndhF* + *rpl32-trnL^UAG^* + *ycf1*.

**Figure 5:**
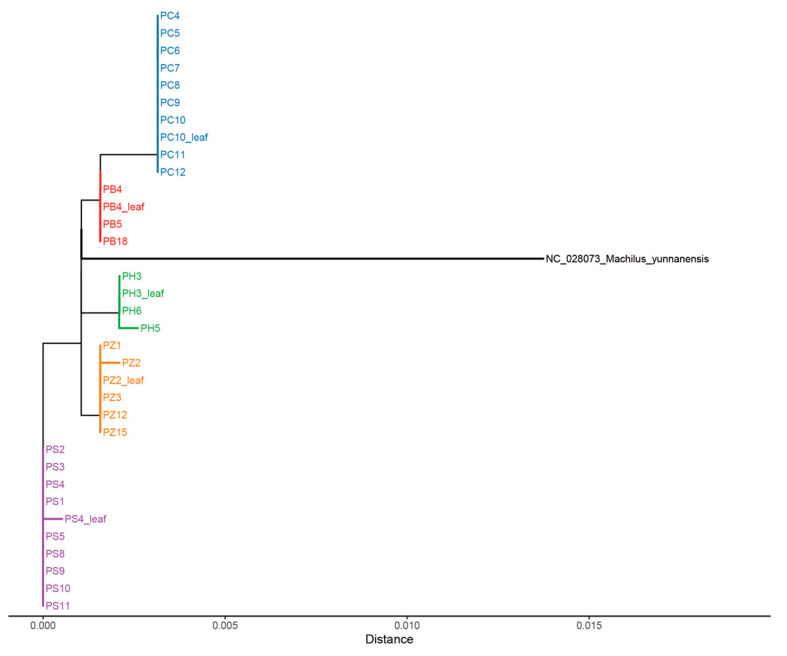
The NJ tree of the *Phoebe*-specific barcode-tested datasets based on *rpl32-trnL^UAG^* + *ycf1,* rooted with *Machilus yunnanensis.* The same colors represent the same species with more than one individual, PB: *P. bournei*, PC: *P. chekiangensis*, PH: *P. hui*, PS: *P. sheareri*, PZ: *P. zhennan*; the leaf samples are indicated by “_leaf”, and other samples are from wood.

NJ phylogenetic analyses (Supplementary Figure S2a–d) show that all the single locus barcodes fail to distinguish between *P. bournei*, *P. chekiangensis*, *P. hui*, *P. sheareri*, and *P. zhennan* completely. In contrast, these five target species are well discriminated when *rpl32-trnL^UAG^* is combined with *ycf1* and combined with *ycf1* and Ψ*ycf1-ndhF* (Figure 4a,b and Figure 5). Furthermore, these two combinations both identify ten other species in the genus of *Phoebe*, leaving only five highly unidentified species remain. This species-level success rate aligns well with the relatively high IRS at the individual level (Table 1). The multi-locus combinations of *petA-psbJ-psbL-psbF-psbE* + *rpl32-trnL^UAG^* + *ycf1* and *petA-psbJ-psbL-psbF-psbE* + Ψ*ycf1-ndhF* + *rpl32-trnL^UAG^* + *ycf1* have even higher IRS (Table 1); however, they do not yield a satisfactory identification result for *P. bournei*, *P. chekiangensis*, *P. hui*, *P. sheareri*, and *P. zhennan*, although these combinations can distinguish 16 tree species (Supplementary Figure S3). In addition, the samples we collected confirmed that *rpl32-trnL^UAG^* + *ycf1* can effectively distinguish the five target species (Figure 5) and implied that DNA from both the wood and the leaves is the same.

## 3. Discussion

In this study, we show that two sets of combined hotspot regions—*rpl32*-*trnL^UAG^* + *ycf1* and Ψ*ycf1*-*ndhF + rpl32*-*trnL^UAG^* + *ycf1*—can accurately distinguish the five endangered *Phoebe* species (*P. bournei*, *P. chekiangensis*, *P. hui*, *P. sheareri*, and *P. zhennan*). This result is supported by the NJ tree analyses on barcodes extracted from the genome sequences of 20 *Phoebe* species (Figure 4a,b), but also by the wood and leaf samples of the five species (Figure 5). The perfect clustering of the same species from multiple sites in Southern China further indicates that these two-locus combinations are geographically stable and consistent (Figure 5). Moreover, the close genetic relationship of *P. bournei*, *P. chekiangensis*, *P. hui*, *P. sheareri*, and *P. zhennan*, in general, aligns well with the genetic relationship based on plastid genomes of Lauraceae species [1,8,34]. This confirms the robustness of these two-locus combinations for differentiating the five species.

Although the mean pi value of *ycf1* accounts for half the magnitude of the peak of *petA*-*psbJ*-*psbL*-*psbF*-*psbE* (Figure 3), earlier studies have suggested that *ycf1* likely represents the most variable region compared to standard barcodes such as *rbcL*, *matK*, *trnH*-*psbA*, and *ITS* [19,30,35,36,37,38,39,40,41,42,43,44,45]. We find that *ycf1* can identify three target *Phoebe* species (*P. chekiangensis*, *P. hui,* and *P. sheareri*) and nine other *Phoebe* species among the twenty *Phoebe* species (Supplementary Figure S2d). This single-locus barcode still fails to differentiate the five endangered *Phoebe* species. Between the two *ycf1*-based combinations leading to a successful identification of the target species, *ycf1* and *rpl32*-*trnL*^UAG^ is more cost-effective than *ycf1* combined with Ψ*ycf1-ndhF* and *rpl32*-*trnL*^UAG^ [46], while retaining the same ISR (Table 1). *rpl32*-*trnL^UAG^* + *ycf1* can additionally identify 10 *Phoebe* species tested in this study (Figure 4a). Furthermore, the amplification rates of *ycf1* and *rpl32*-*trnL^UAG^* reached 93.94% and 87.88% (Supplementary Table S3), respectively, exceeding a commonly accepted rate of 70% [47]. This suggests that the combination of *ycf1* and *rpl32*-*trnL^UAG^* can serve as a reliable and realistic marker for the future identification of endangered *Phoebe* species.

The NJ tree analysis confirms the genetic similarity between wood and leaf tissues (Figure 5), which is consistent with previous studies on species such as *Lithocarpus* [48], *Aquilaria* [49,50], and *Pterocarpus* [51]. The experimental findings of our study demonstrate identical sequences in wood and leaf samples from five *Phoebe* species, amplified by two markers. The genetic consistency observed between leaves and wood arises from their developmental origins, which begin with protoplasts that differentiate into distinct plastids—chloroplasts in leaves and amyloslasts in wood. Despite these functional differences, the plastids retain identical genetic information [52,53]. This genetic uniformity supports the application of molecular markers for species identification and origin verification in both plant and woody tissues, aligning with previous findings in plant biology [25].

Although the information obtained from plant and woody tissues is consistent, extracting DNA from different plant tissues remains challenging. This depends on several factors, including the low proportion of parenchyma cells in wood tissues [1,54,55,56], which are the primary source of DNA. Another factor is the degradation of DNA due to programmed cell death and environmental factors [49,55,57,58]. In this study, DNA was efficiently extracted from modern wood by our pre-treating methods; however, extracting DNA from archeological wood remains difficult [1,59]. Jiao [1] developed a targeted cell DNA extraction method that enables DNA recovery from a wide range of wood sources. This offers a promising approach for extracting DNA from recalcitrant plant tissues, such as those rich in secondary metabolites or stored for long periods. Alternative amplification strategies, such as two-stage PCR [60], have also been suggested to improve the sensitivity and efficiency of detecting DNA in degraded samples. Together, these developments have expanded the toolkit for wood identification and forensic analysis. When combined with the specific primers used in this study, these tools can effectively differentiation *Phoebe* species.

## 4. Materials and Methods

### 4.1. Sampling of Materials

**Figure 6:**
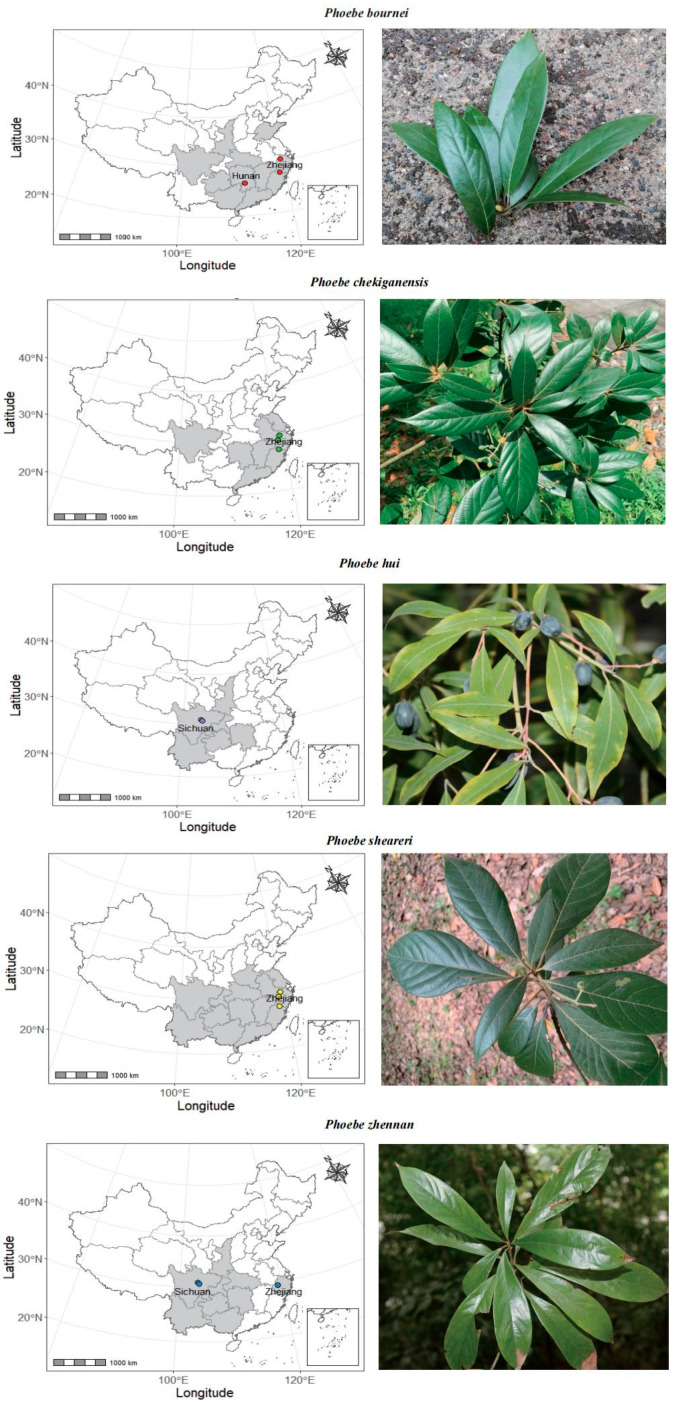
Sampling sites and leaf of five *Phoebe* species. Colored points indicate the location of samples and shaded areas show province-level distribution of corresponding species according to IPLANT (https://www.iplant.cn/).

We collected wood and leaf materials from mature trees (20~130 years) of the five *Phoebe* species in the Sichuan and Zhejiang provinces, southern China (Figure 6). Since trees of the five species are frequently ancient and protected, it is difficult to collect a large amount of wood material from the tree stem. Thus, the majority of wood samples were either from tree cores (using an increment borer) or large branches, which do not visually show any evidence of decay. Heartwood was used when present in wood samples as this is more frequently used for furniture manufacturing than sapwood, while sapwood was only used when heartwood was not available. In total, 33 wood (one for each of the 33 trees) and 5 leaf samples (one per species) were collected from the five species (see details in Supplementary Table S4). While the leaves were stored in a separate Ziplock in a refrigerator at −20 °C, wood materials were air dried at room temperature (~20 °C).

### 4.2. Candidate Divergent Hotspot Regions

Although we targeted the five aforementioned species, we first acquired 55 plastomes from a total of 20 *Phoebe* species endemic to China (Supplementary Table S2; 2–6 sequences per species) from the NCBI platform. This approach helps to identify hotspot regions of divergent sequences that can be used to design primers for PCR markers able to distinguish as many of China’s native *Phoebe* species as possible. Structural features of these plastomes, including GC content, the count of various types of gene, and the length of different regions, were analyzed using OGDRAW software (Draw Organelle Genome Maps) [61]. The inverted repeat (IR) expansion and contraction [62] were detected from one sample per species using the IRscope program (https://irscope.shinyapps.io/irapp/). The candidate divergent hotspot regions were then identified according to the nucleotide diversity (Pi) of the 55 sequences. The Pi values were calculated using a sliding window method using the DnaSP v.5.10.01 software [63], with a window length of 800 sites and a step size of 100 sites. Species-specific primer regions were determined based on these Pi values (see Section 2.2).

### 4.3. DNA Extraction

DNA was extracted from wood and leaf samples of the five *Phoebe* species (Section 4.1; Supplementary Table S4). For wood samples, the surface was first removed using razor blades cleaned with 70% (*w*/*w*) ethanol. Then, the remaining fresh wood samples were cut into (~0.5 mm) chips, which were ground to a fine powder using the Mixer Mill MM 400 (Retsch, Haan, Germany, two sequential 3 min grinding cycles at 30 Hz). Leaf samples were homogenized using Precellys Evolution (Bertin Technologies, Montigny-le-Bretonneux, France) with ceramic beads (3 mm diameter). The total DNA of these samples was extracted using the Dneasy Plant Mini Kit (Qiagen, Hilden, Germany) [47,64].

### 4.4. PCR Amplification and Sequencing

To verify the usefulness of the candidate hotspot regions (see Section 2.2) for practical species identification, we chose the *rpl32*-*trnL*^UAG^ and *ycf1* primers (their combination is efficient for differentiation of the five *Phoebe* species) for PCR amplification in 33 samples. PCR amplification was performed in a 20 μL reaction volume, with 2 μL of template DNA (i.e., total DNA) extracted from each sample, 10 μL of 2× Phanta Max Master Mix (Contains Mg^2+^) (Vazyme, Nanjing, China), 0.4 μL of each designed primer (Table 2), and 7.2 µL of ddH_2_O (double distilled water). The mixture was subjected to amplification using a Biometra Trio48 thermal cycler Analytik Jena AG, Jena, Germany) using the following protocol: one cycle of initial denaturation for 2 min at 98 °C; followed by 35 cycles of denaturation for 10 s at 98 °C, annealing for 15 s at 57 °C, and an extension step for 30 s at 72 °C; then, it continued in a final cycle of extension for 5 min at 72 °C. Each PCR reaction underwent separation using 1.5% agarose gels, followed by staining with ethidium bromide and examination under ultraviolet light [65]. All PCR products were sequenced in both directions at the Sangon Biotech (Shanghai, China) Co., Ltd.

The forward and reverse sequences were assembled into single contigs using SeqMan version 7.0.0 (DNASTAR, Madison, WI, USA). To confirm the absence of contamination, all sequences were validated via the BLAST analysis platform. Refs. [66,67] against the NCBI nucleotide database. Sequence alignments for each genetic marker were conducted using MEGA7 [68], followed by manual inspection and trimming to generate final sequence matrices.

### 4.5. Analysis of Phylogeny

We further analyzed the phylogenetic relationships among the 20 *Phoebe* species, including 55 sequences obtained from NCBI. We first compiled sequence matrices for the four single candidate barcodes (see Results) and their combinations. Distance matrices were subsequently calculated using the “ape” R package (version 5.8-1) [69], using the default method described by Kimura (1980) [70]. Finally, neighbor-joining (NJ) trees were constructed using the “bionj” function in “ape” R package. We similarly used this method to build a family tree for new sequences generated from 38 wood and leaf samples of *P. bournei*, *P. chekiangensis*, *P. hui*, *P. sheareri,* and *P. zhennan* (see Section 2.3); additionally, *Machilus yunnanensis* downloaded from the NCBI Nucleotide database (Accession number: NC_028073) was selected as the outgroup.

## 5. Conclusions

This study aimed to investigate the potential of barcoding for identifying the five endangered *Phoebe* species in China, including *P. bournei*, *P. chekiangensis*, *P. hui*, *P. sheareri*, and *P. zhennan*. Although the DNA structures are highly similar, we found that combinations of Ψ*ycf1*-*ndhF* and *rpl32*-*trnL^UAG^* and *ycf1* can differentiate the five target as well as ten other species in the *Phoebe* genus, with the same ISR of 70.91%. We also validated the robustness of the *ycf1* and *rpl32*-*trnL^UAG^* combination using wood and leaf samples of the five target species collected from multiple sites in China, indicating that the two loci are stable between plant organs and across populations. The other theoretical combination, despite not being tested using newly measured data, may also be used as a reliable barcode. However, this three-locus barcode did not substantially improve the species resolution and is more labor- and cost-intensive than the two-locus barcode. The DNA barcoding technique bridges a critical gap in the quick and accurate identification of *Phoebe* species in future plant protection and wood trade. Specific barcoding for other *Phoebe* species still requires future investigation.

## Figures and Tables

**Table 1:** Intra- and inter-specific genetic distance (Material and Methods) and identification success rate (ISR) for the four candidate loci and their combinations. Min. and Max. represent minimum and maximum distances, respectively. P: *petA-psbJ-psbL-psbF-psbE*; F: Ψ*ycf1-ndhF*; R: *rpl32-trnL^UAG^*; Y: *ycf1*.

DNA Barcodes	Intraspecific Distance			Interspecific Distance			ISR
	Min	Max	Mean	Min	Max	Mean	
P	0.0000	0.0083	0.0011	0.0000	0.0077	0.0007	16.36%
R	0.0000	0.0000	0.0000	0.0000	0.0109	0.0015	40.00%
Y	0.0000	0.0010	0.0002	0.0000	0.0106	0.0012	56.36%
F	0.0000	0.0030	0.0005	0.0000	0.0089	0.0013	67.27%
P + R	0.0000	0.0045	0.0006	0.0000	0.0102	0.0014	50.91%
P + Y	0.0000	0.0042	0.0007	0.0000	0.0096	0.0014	69.09%
P + F	0.0000	0.0048	0.0007	0.0000	0.0075	0.0009	49.09%
R + Y	0.0000	0.0005	0.0001	0.0000	0.0112	0.0015	70.91%
F + R	0.0000	0.0006	0.0001	0.0000	0.0087	0.0011	49.09%
F + Y	0.0000	0.0011	0.0002	0.0000	0.0091	0.0010	56.36%
P + R + Y	0.0000	0.0029	0.0005	0.0000	0.0110	0.0016	78.18%
P + F + R	0.0000	0.0032	0.0005	0.0000	0.0094	0.0013	65.45%
P + F + Y	0.0000	0.0031	0.0005	0.0000	0.0090	0.0012	63.64%
F + R + Y	0.0000	0.0007	0.0001	0.0000	0.0097	0.0013	70.91%
P + F + R + Y	0.0000	0.0023	0.0004	0.0000	0.0102	0.0014	78.18%

**Table 2:** *Phoebe* primers used for amplification.

*Phoebe* Primers	Type	Primer Sequences (5′–3′)
*rpl32*-*trnL*^UAG^	Forward	GCGAGATGGGGGTTGTAACT
	Reverse	AGTATCATGGCAGGGGGTCA
*ycf1*	Forward	TGACCCCTTAACCAGTTTTTCCA
	Reverse	CTGAAACCCTGGCGCAAATC

## Data Availability

The original contributions presented in this study are included in the article/Supplementary Materials. Further inquiries can be directed to the corresponding authors.

## Supplementary Materials

The following supporting information can be downloaded at https://www.mdpi.com/article/10.3390/plants14182895/s1. Supplementary Material S1: Figure S1: Gene maps of plastomes of 20 species of *Phoebe*. Figure S2: Neighbor-joining (NJ) trees based on multi loci. Figure S3: Neighbor-joining (NJ) trees based on multi loci: (a) P + F + R (b) P + F + Y (c) P + R + Y (d) P + F + R + Y. P: *petA-psbJ-psbL-psbF-psbE*; F: Ψ*ycf1-ndhF*; R:*rpl32-trnLUAG*; Y*:ycf1*. Table S1: Summary of *Phoebe* species included in two lists. Table S2: Characteristic of chloroplast genomes of *Phoebe*. Table S3: The 33 samples used to test primer (*rpl32*-*trnL*^UAG^, *ycf1*) universality. Table S4: Samples source information. Supplementary Material S2: Sequence identity plots among 55 *Phoebe* species.

## References

[1] Jiao L., Lu Y., Zhang M., Chen Y., Wang Z., Guo Y., Xu C., Guo J., He T., Ma L. (2022). Ancient Plastid Genomes Solve the Tree Species Mystery of the Imperial Wood “Nanmu” in the Forbidden City, the Largest Existing Wooden Palace Complex in the World. Plants People Planet.

[2] Ding X., Xiao J.H., Li L., Conran J.G., Li J. (2019). Congruent Species Delimitation of Two Controversial Gold-thread Nanmu Tree Species Based on Morphological and Restriction Site-associated DNA Sequencing Data. J. Syst. Evol..

[3] Lan Y. (1994). Imperial Wood Procurement in Ming and Qing Dynasties. Hist. Res..

[4] (2023). IUCN, The Iucn Red List of Threatened Species. https://www.iucnredlist.org.

[5] (2021). National Forestry and Grassland Administration, List of National Key Protected Wild Plants in China. https://www.forestry.gov.cn/c/www/gkml/11057.jhtml.

[6] Liu J., Lindenmayer D.B., Yang W., Ren Y., Campbell M.J., Wu C., Luo Y., Zhong L., Yu M. (2019). Diversity and Density Patterns of Large Old Trees in China. Sci. Total Environ..

[7] Liu Z.F., Ci X.Q., Li L., Li H.-W., Conran J.G., Li J. (2017). DNA Barcoding Evaluation and Implications for Phylogenetic Relationships in Lauraceae from China. PLoS ONE.

[8] Liu Z.F., Ma H., Ci X.Q., Li L., Song Y., Liu B., Li H.W., Wang S.L., Qu X.J., Hu J.L. (2021). Can Plastid Genome Sequencing Be Used for Species Identification in Lauraceae?. Bot. J. Linn. Soc..

[9] Yang S., Huang J., Qu Y., Zhang D., Tan Y., Wen S., Song Y. (2024). Phylogenetic Incongruence in an Asiatic Species Complex of the Genus Caryodaphnopsis (Lauraceae). BMC Plant Biol..

[10] Hebert P.D.N., Cywinska A., Ball S.L., deWaard J.R. (2003). Biological Identifications through DNA Barcodes. Proc. R. Soc. Lond. B.

[11] Julia S., Soepadmo E., Yahud W. (2009). Problem in the Generic Delimitation between *Alseodaphne*, *Dehaasia* and *Nothaphoebe* (*Lauraceae*) in Borneo. Blumea-Biodivers. Evol. Biogeogr. Plants.

[12] Li L., Li J., Rohwer J.G., Van Der Werff H., Wang Z., Li H. (2011). Molecular Phylogenetic Analysis of the *Persea* Group (Lauraceae) and Its Biogeographic Implications on the Evolution of Tropical and Subtropical Amphi-pacific Disjunctions. Am. J. Bot..

[13] Song Y., Yu W., Tan Y., Jin J., Wang B., Yang J., Liu B., Corlett R.T. (2020). Plastid Phylogenomics Improve Phylogenetic Resolution in the Lauraceae. J. Syst. Evol..

[14] Van Der Werff H., Richter H.G. (1996). Toward an Improved Classification of Lauraceae. Ann. Mo. Bot. Gard..

[15] Xu B., Zhu T., Li J., Liu S. (2013). Identification of Wood between *Phoebe Zhennan* and *Machilus Pingii* Using the Gas Chromatography-Mass Spectrometry Direct Injection Technique. Eur. J. Mass. Spectrom..

[16] Li Y., Xu W., Zou W., Jiang D., Liu X. (2017). Complete Chloroplast Genome Sequences of Two Endangered *Phoebe* (Lauraceae) Species. Bot. Stud..

[17] Liu C., Chen H.H., Tang L.Z., Khine P.K., Han L.H., Song Y., Tan Y.H. (2022). Plastid Genome Evolution of a Monophyletic Group in the Subtribe Lauriineae (Laureae, Lauraceae). Plant Divers..

[18] Shi W., Song W., Chen Z., Cai H., Gong Q., Liu J., Shi C., Wang S. (2023). Comparative Chloroplast Genome Analyses of Diverse *Phoebe* (Lauraceae) Species Endemic to China Provide Insight into Their Phylogeographical Origin. PeerJ.

[19] Song Y., Yao X., Tan Y., Gan Y., Yang J., Corlett R.T. (2017). Comparative Analysis of Complete Chloroplast Genome Sequences of Two Subtropical Trees, *Phoebe Sheareri* and *Phoebe Omeiensis* (Lauraceae). Tree Genet. Genomes.

[20] Liu B., Yang Y., Ferguson D.K. (2022). Phylogeny and Taxonomy of *Cinnamomum* (Lauraceae). Ecol. Evol..

[21] Cai C., Ma H., Ci X., Conran J.G., Li J. (2021). Comparative Phylogenetic Analyses of Chinese *Horsfieldia* (Myristicaceae) Using Complete Chloroplast Genome Sequences. J. Syst. Evol..

[22] Tian Y., Zhou J., Zhang Y., Wang S., Wang Y., Liu H., Wang Z. (2021). Research Progress in Plant Molecular Systematics of Lauraceae. Biology.

[23] Xiong Y., Xiong Y., Jia S., Ma X. (2020). The Complete Chloroplast Genome Sequencing and Comparative Analysis of Reed Canary Grass (*Phalaris arundinacea*) and Hardinggrass (*P. aquatica*). Plants.

[24] Li X., Yang Y., Henry R.J., Rossetto M., Wang Y., Chen S. (2015). Plant DNA Barcoding: From Gene to Genome. Biol. Rev..

[25] Lowe A.J., Cross H.B. (2011). The Application of DNA Methods to Timber Tracking and Origin Verificat Ion. IAWA J..

[26] Hajibabaei M., Janzen D.H., Burns J.M., Hallwachs W., Hebert P.D.N. (2006). DNA Barcodes Distinguish Species of Tropical Lepidoptera. Proc. Natl. Acad. Sci. USA.

[27] Hu J., Ci X., Liu Z., Dormontt E.E., Conran J.G., Lowe A.J., Li J. (2022). Assessing Candidate DNA Barcodes for Chinese and Internationally Traded Timber Species. Mol. Ecol. Resour..

[28] Lendvay B., Hartmann M., Brodbeck S., Nievergelt D., Reinig F., Zoller S., Parducci L., Gugerli F., Büntgen U., Sperisen C. (2018). Improved Recovery of Ancient DNA from Subfossil Wood—Application to the World’s Oldest Late Glacial Pine Forest. New Phytol..

[29] Chen Z., Gao L., Wang H., Feng S. (2024). Molecular Identification and Phylogenetic Analysis of *Cymbidium* Species (Orchidaceae) Based on the Potential DNA Barcodes *matK*, *rbcL*, *psbA*-*trnH*, and Internal Transcribed Spacer. Agronomy.

[30] Li D.Z., Gao L.M., Wang H., Ge X.J., Liu J.Q., Chen Z.D., Zhou S.L., Chen S.L., Yang J.B., China Plant BOL Group (2011). Comparative Analysis of a Large Dataset Indicates That Internal Transcribed Spacer (*ITS*) Should Be Incorporated into the Core Barcode for Seed Plants. Proc. Natl. Acad. Sci. USA.

[31] Du Z., Qimike A., Yang C., Chen J., Wang Q. (2011). Testing Four Barcoding Markers for Species Identification of Potamogetonaceae. J. Syst. Evol..

[32] Fu Y., Jiang W., Fu C. (2011). Identification of Species within *Tetrastigma* (Miq.) Planch. (Vitaceae) Based on DNA Barcoding Techniques. J. Syst. Evol..

[33] Li L., Li J., Li X.W. (2011). Taxonomic Revision of Five Species of the Genus *Phoebe* (Lauraceae) from China. Plant Divers..

[34] Xu J., Zhang H., Yang F., Zhu W., Li Q., Cao Z., Song Y., Xin P. (2025). Phylogeny of *Camphora* and *Cinnamomum* (Lauraceae) Based on Plastome and Nuclear Ribosomal DNA Data. Int. J. Mol. Sci..

[35] Chen S., Yao H., Han J., Liu C., Song J., Shi L., Zhu Y., Ma X., Gao T., Pang X. (2010). Validation of the *ITS2* Region as a Novel DNA Barcode for Identifying Medicinal Plant Species. PLoS ONE.

[36] Ford C.S., Ayres K.L., Toomey N., Haider N., Van Alphen Stahl J., Kelly L.J., Wikström N., Hollingsworth P.M., Duff R.J., Hoot S.B. (2009). Selection of Candidate Coding DNA Barcoding Regions for Use on Land Plants. Bot. J. Linn. Soc..

[37] Hernández-León S., Gernandt D.S., Pérez De La Rosa J.A., Jardón-Barbolla L. (2013). Phylogenetic Relationships and Species Delimitation in *Pinus* Section *Trifoliae* Inferrred from Plastid DNA. PLoS ONE.

[38] Kress W.J., Wurdack K.J., Zimmer E.A., Weigt L.A., Janzen D.H. (2005). Use of DNA Barcodes to Identify Flowering Plants. Proc. Natl. Acad. Sci. USA.

[39] Kress W.J., Erickson D.L., Jones F.A., Swenson N.G., Perez R., Sanjur O., Bermingham E. (2009). Plant DNA Barcodes and a Community Phylogeny of a Tropical Forest Dynamics Plot in Panama. Proc. Natl. Acad. Sci. USA.

[40] Little D.P., Knopf P., Schulz C. (2013). DNA Barcode Identification of Podocarpaceae—The Second Largest Conifer Family. PLoS ONE.

[41] Pang X., Liu C., Shi L., Liu R., Liang D., Li H., Cherny S.S., Chen S. (2012). Utility of the *trnH*–*psbA* Intergenic Spacer Region and Its Combinations as Plant DNA Barcodes: A Meta-Analysis. PLoS ONE.

[42] Yao H., Song J., Liu C., Luo K., Han J., Li Y., Pang X., Xu H., Zhu Y., Xiao P. (2010). Use of *ITS2* Region as the Universal DNA Barcode for Plants and Animals. PLoS ONE.

[43] Zhang C., Wang F., Yan H., Hao G., Hu C., Ge X. (2012). Testing DNA Barcoding in Closely Related Groups of *Lysimachia* L. (Myrsinaceae). Mol. Ecol. Resour..

[44] Wang X.C., Liu C., Huang L., Bengtsson-Palme J., Chen H., Zhang J.H., Cai D., Li J.Q. (2015). ITS1: A DNA Barcode Better than ITS2 in Eukaryotes?. Mol. Ecol. Resour..

[45] Dong W., Cheng T., Li C., Xu C., Long P., Chen C., Zhou S. (2014). Discriminating Plants Using the DNA Barcode *rbcL*b: An Appraisal Based on a Large Data Set. Mol. Ecol. Resour..

[46] Group C.P.W. (2009). A DNA Barcode for Land Plants. Proc. Natl. Acad. Sci. USA.

[47] Yu M., Jiao L., Guo J., Wiedenhoeft A.C., He T., Jiang X., Yin Y. (2017). DNA Barcoding of Vouchered Xylarium Wood Specimens of Nine Endangered *Dalbergia* Species. Planta.

[48] Cannon C.H., Manos P.S. (2003). Phylogeography of the Southeast Asian Stone Oaks (*Lithocarpus*). J. Biogeogr..

[49] Jiao L., Yin Y., Cheng Y., Jiang X. (2014). DNA Barcoding for Identification of the Endangered Species *Aquilaria Sinensis*: Comparison of Data from Heated or Aged Wood Samples. Holzforschung.

[50] Tanaka S., Ito M. (2020). DNA Barcoding for Identification of Agarwood Source Species Using *trnL*-*trnF* and *matK* DNA Sequences. J. Nat. Med..

[51] Jiao L., Yu M., Wiedenhoeft A.C., He T., Li J., Liu B., Jiang X., Yin Y. (2018). DNA Barcode Authentication and Library Development for the Wood of Six Commercial *Pterocarpus* Species: The Critical Role of Xylarium Specimens. Sci. Rep..

[52] Ohyama M., Baba K., Itoh T. (2001). Wood Identification of Japanese *Cyclobalanopsis* Species (Fagaceae) Based on DNA Polymorphism of the Intergenic Spacer between *trnT* and *trnL* 5′ Exon. J. Wood Sci..

[53] Pizzolato T.D. (1978). A Tannic Acid-Ferric Chloride-Toluidine Blue Stain for Wood Amyloplasts Embedded in Epoxy Resin. For. Sci..

[54] Deguilloux M., Pemonge M., Petit R. (2002). Novel Perspectives in Wood Certification and Forensics: Dry Wood as a Source of DNA. Proc. R. Soc. Lond. B.

[55] Jiao L., Lu Y., He T., Guo J., Yin Y. (2020). DNA Barcoding for Wood Identification: Global Review of the Last Decade and Future Perspective. IAWA J..

[56] Winter H., Robinson D.G., Heldt H.W. (1994). Subcellular Volumes and Metabolite Concentrations in Spinach Leaves. Planta.

[57] Abe H., Watanabe U., Yoshida K., Kuroda K., Zhang C. (2011). Changes in Organelle and DNA Quality, Quantity, and Distribution in the Wood of *Cryptomeria Japonica* over Long-Term Storage. IAWA J..

[58] Jiao L., Yin Y., Xiao F., Sun Q., Song K., Jiang X. (2012). Comparative Analysis of Two DNA Extraction Protocols from Fresh and Dried Wood of *Cunninghamia Lanceolata* (Taxodiaceae). IAWA J..

[59] Lu Y., Jiao L., Sun G., Wang J., Liu S., Li R., Zhang Y., Guo Y., Guo J., Jiang X. (2023). Preservation Status and Microbial Community of Waterlogged Archaeological Woods over 7800 Years Old at the Jingtoushan Site, China. Wood Sci. Technol..

[60] Rogers S.O., Kaya Z. (2006). DNA from Ancient Cedar Wood from King Midas’ Tomb, Turkey, and al-Aksa Mosque, Israel. Silvae Genet..

[61] Greiner S., Lehwark P., Bock R. (2019). OrganellarGenomeDRAW (OGDRAW) Version 1.3.1: Expanded Toolkit for the Graphical Visualization of Organellar Genomes. Nucleic Acids Res..

[62] Amiryousefi A., Hyvönen J., Poczai P. (2018). IRscope: An Online Program to Visualize the Junction Sites of Chloroplast Genomes. Bioinformatics.

[63] Librado P., Rozas J. (2009). DnaSP v5: A Software for Comprehensive Analysis of DNA Polymorphism Data. Bioinformatics.

[64] Lee S.Y., Ng W.L., Mahat M.N., Nazre M., Mohamed R. (2016). DNA Barcoding of the Endangered *Aquilaria* (Thymelaeaceae) and Its Application in Species Authentication of Agarwood Products Traded in the Market. PLoS ONE.

[65] Rachmayanti Y., Leinemann L., Gailing O., Finkeldey R. (2006). Extraction, Amplification and Characterization of Wood DNA from Dipterocarpaceae. Plant Mol. Biol. Rep..

[66] Altschul S.F., Gish W., Miller W., Myers E.W., Lipman D.J. (1990). Basic Local Alignment Search Tool. J. Mol. Biol..

[67] Camacho C., Boratyn G.M., Joukov V., Vera Alvarez R., Madden T.L. (2023). ElasticBLAST: Accelerating Sequence Search via Cloud Computing. BMC Bioinform..

[68] Kumar S., Stecher G., Tamura K. (2016). MEGA7: Molecular Evolutionary Genetics Analysis Version 7.0 for Bigger Datasets. Mol. Biol. Evol..

[69] Paradis E., Claude J., Strimmer K. (2004). APE: Analyses of Phylogenetics and Evolution in R Language. Bioinformatics.

[70] Kimura M. (1980). A Simple Method for Estimating Evolutionary Rates of Base Substitutions through Comparative Studies of Nucleotide Sequences. J. Mol. Evol..

