# Supplementary material for: DNA Barcoding Provides Taxonomic Clues for Identifying Five Endangered Phoebe Species in Southern China
Source: Plants (Basel). 2025 Sep 18;14(18):2895. doi: 10.3390/plants14182895 (PMC12473773; doi:10.3390/plants14182895)
Supplement: Supplementary file 1 [file plants-14-02895-s001.zip › Supplementary material 1.pdf]

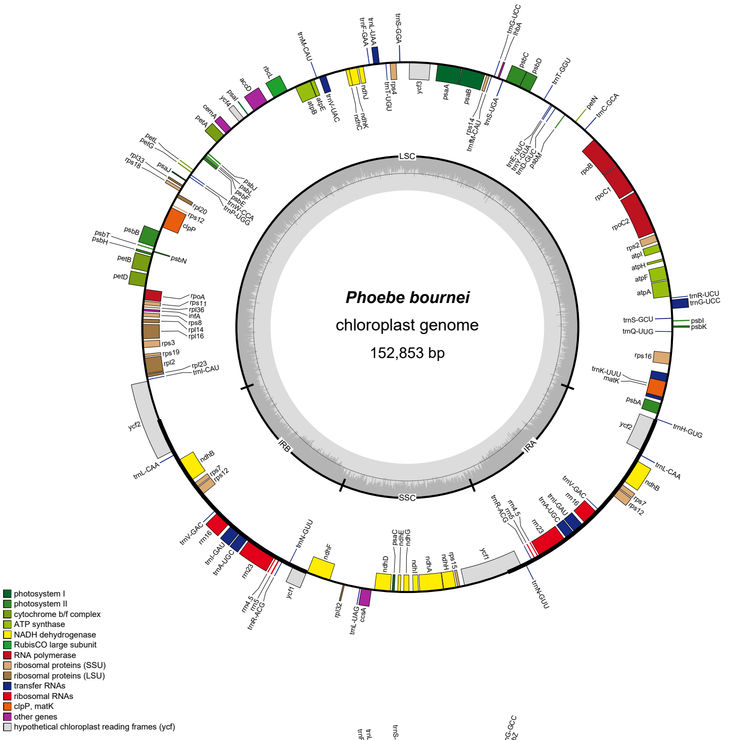
LSC
Phoebe bournei
chloroplast genome
152,853 bp
IRA
SSC
IRB
photosystem I
photosystem II
cytochrome b/f complex
ATP synthase
NADH dehydrogenase
RubisCO large subunit
RNA polymerase
ribosomal proteins (SSU)
ribosomal proteins (LSU)
transfer RNAs
ribosomal RNAs
clpP, matK
other genes
hypothetical chloroplast reading frames (ycf)

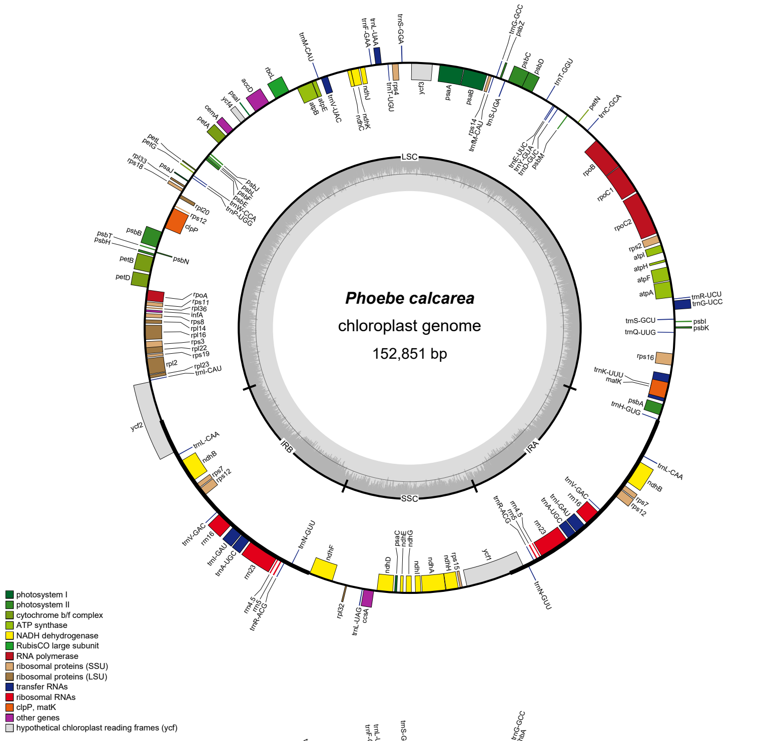
LSC
Phoebe calcarea
chloroplast genome
152,851 bp
IRA
SSC
IRB
photosystem I
photosystem II
cytochrome b/f complex
ATP synthase
NADH dehydrogenase
RubisCO large subunit
RNA polymerase
ribosomal proteins (SSU)
ribosomal proteins (LSU)
transfer RNAs
ribosomal RNAs
clpP, matK
other genes
hypothetical chloroplast reading frames (ycf)

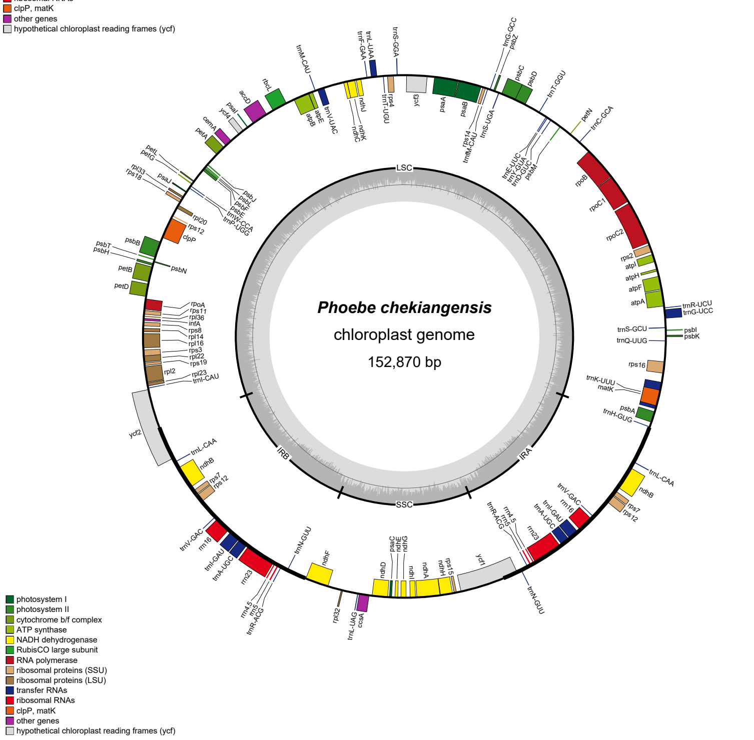
LSC
Phoebe chekiangensis
chloroplast genome
152,870 bp
IRA
SSC
IRB
photosystem I
photosystem II
cytochrome b/f complex
ATP synthase
NADH dehydrogenase
RubisCO large subunit
RNA polymerase
ribosomal proteins (SSU)
ribosomal proteins (LSU)
transfer RNAs
ribosomal RNAs
clpP, matK
other genes
hypothetical chloroplast reading frames (ycf)

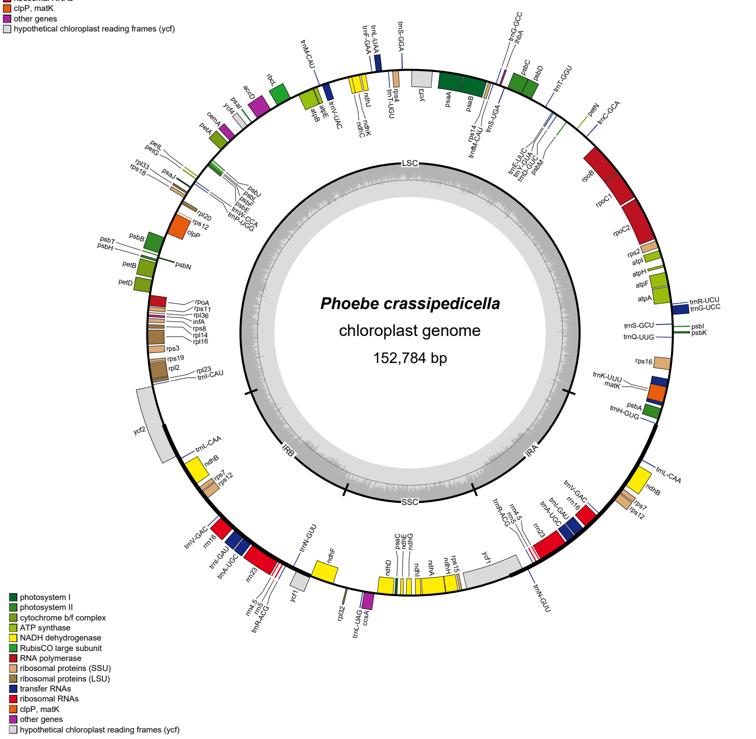
LSC
Phoebe crassipedicella
chloroplast genome
152,784 bp
IRA
SSC
IRB
photosystem I
photosystem II
cytochrome b/f complex
ATP synthase
NADH dehydrogenase
RubisCO large subunit
RNA polymerase
ribosomal proteins (SSU)
ribosomal proteins (LSU)
transfer RNAs
ribosomal RNAs
clpP, matK
other genes
hypothetical chloroplast reading frames (ycf)

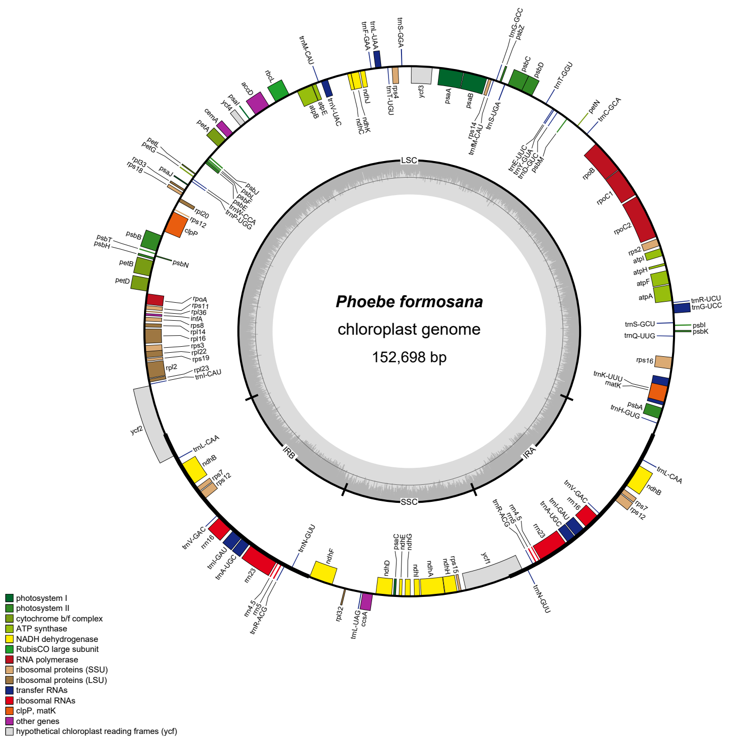
LSC
Phoebe formosana
chloroplast genome
152,698 bp
IRA
SSC
IRB
photosystem I
photosystem II
cytochrome b/f complex
ATP synthase
NADH dehydrogenase
RubisCO large subunit
RNA polymerase
ribosomal proteins (SSU)
ribosomal proteins (LSU)
transfer RNAs
ribosomal RNAs
clpP, matK
other genes
hypothetical chloroplast reading frames (ycf)

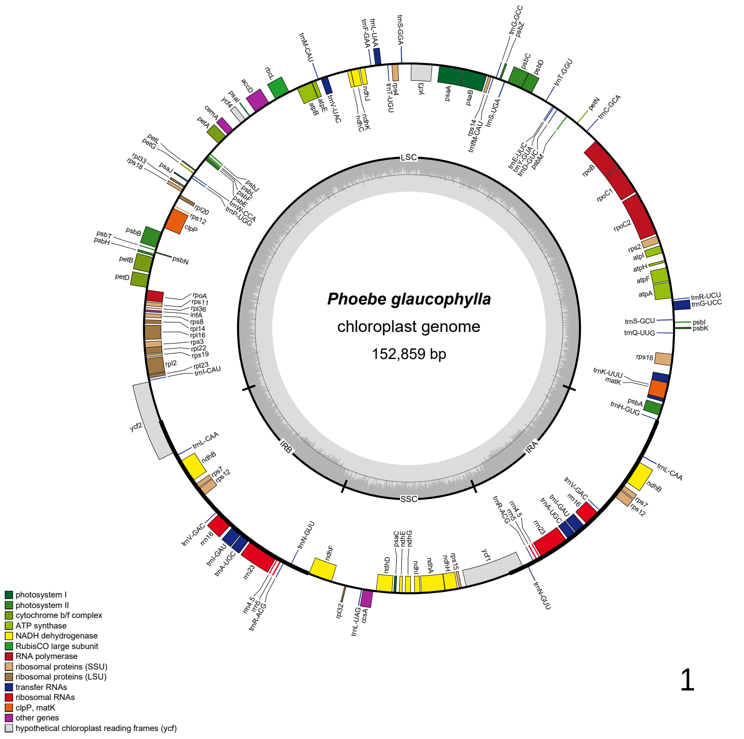
LSC
Phoebe glaucophylla
chloroplast genome
152,859 bp
IRA
SSC
IRB
photosystem I
photosystem II
cytochrome b/f complex
ATP synthase
NADH dehydrogenase
RubisCO large subunit
RNA polymerase
ribosomal proteins (SSU)
ribosomal proteins (LSU)
transfer RNAs
ribosomal RNAs
clpP, matK
other genes
hypothetical chloroplast reading frames (ycf)

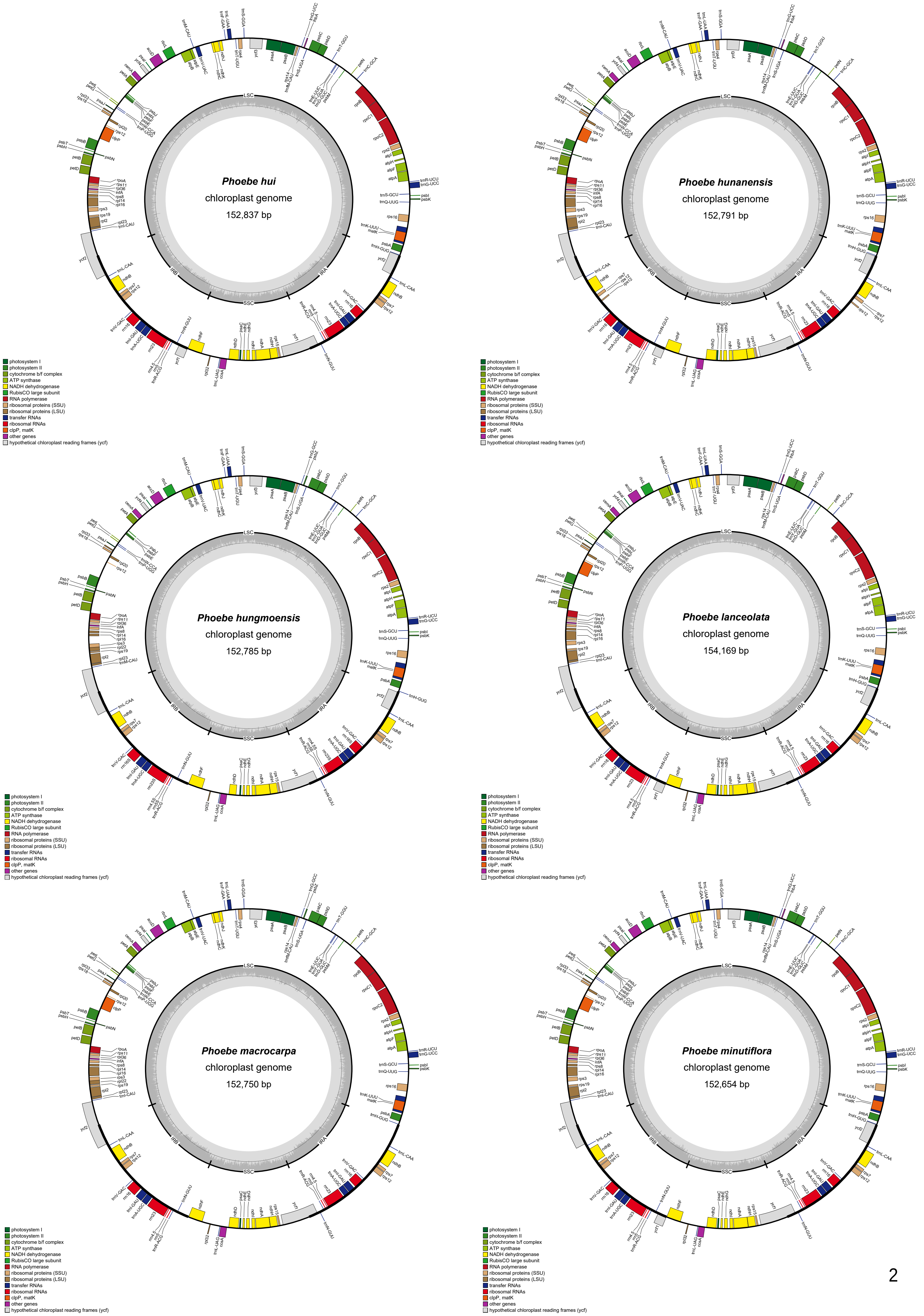
Phoebe hui
chloroplast genome
152,837 bp
Phoebe hunanensis
chloroplast genome
152,791 bp
Phoebe hungmoensis
chloroplast genome
152,785 bp
Phoebe lanceolata
chloroplast genome
154,169 bp
Phoebe macrocarpa
chloroplast genome
152,750 bp
Phoebe minutiflora
chloroplast genome
152,654 bp
LSC
IRA
SSC
IRB
photosystem I
photosystem II
cytochrome b/f complex
ATP synthase
NADH dehydrogenase
RubisCO large subunit
RNA polymerase
ribosomal proteins (SSU)
ribosomal proteins (LSU)
transfer RNAs
ribosomal RNAs
clpP, matK
other genes
hypothetical chloroplast reading frames (ycf)

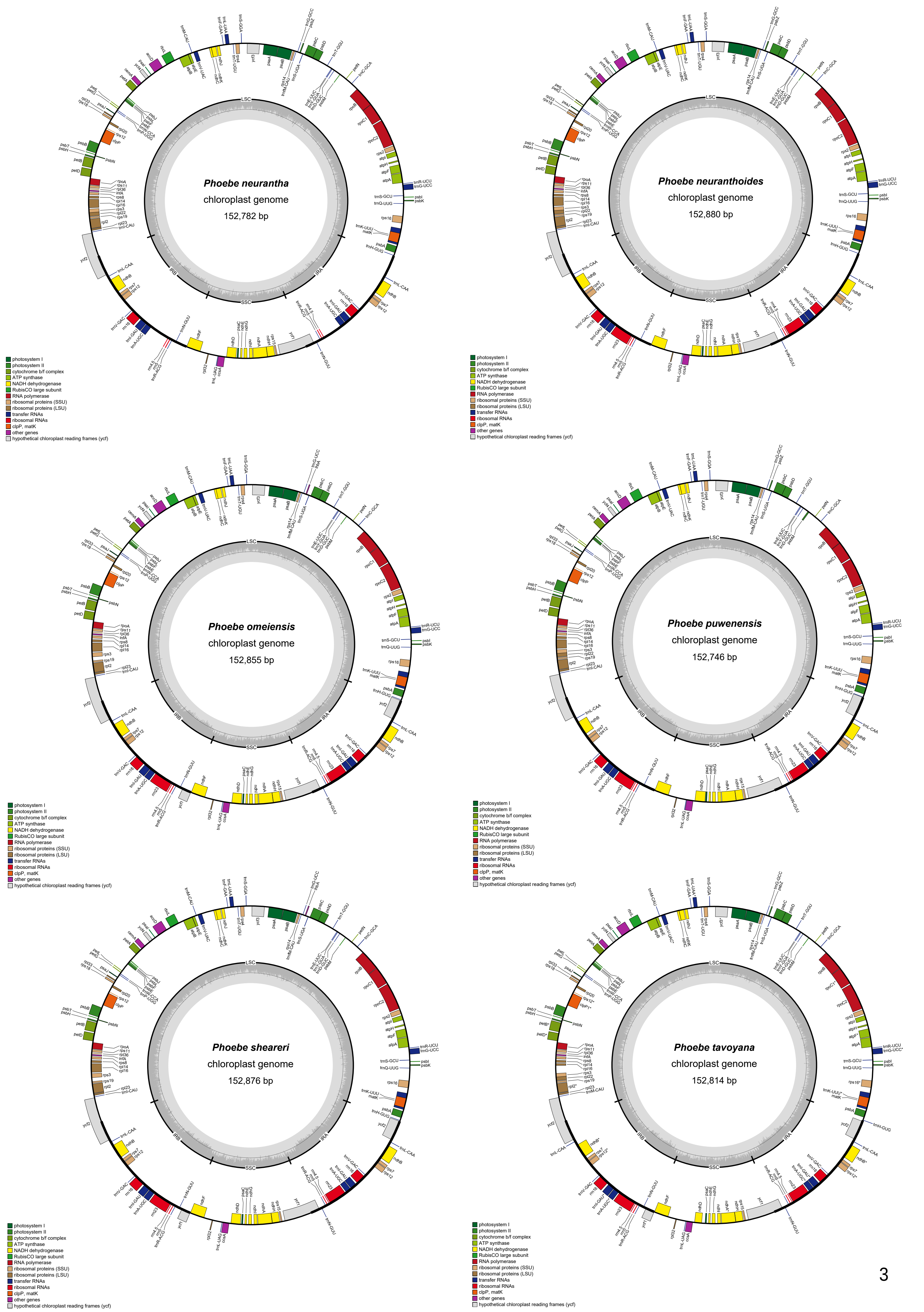
Phoebe neurantha
chloroplast genome
152,782 bp
Phoebe neuranthoides
chloroplast genome
152,880 bp
Phoebe omeiensis
chloroplast genome
152,855 bp
Phoebe puwenensis
chloroplast genome
152,746 bp
Phoebe sheareri
chloroplast genome
152,876 bp
Phoebe tavoyana
chloroplast genome
152,814 bp
LSC
SSC
IRA
IRB
photosystem I
photosystem II
cytochrome b/f complex
ATP synthase
NADH dehydrogenase
RubisCO large subunit
RNA polymerase
ribosomal proteins (SSU)
ribosomal proteins (LSU)
transfer RNAs
ribosomal RNAs
clpP, matK
other genes
hypothetical chloroplast reading frames (ycf)

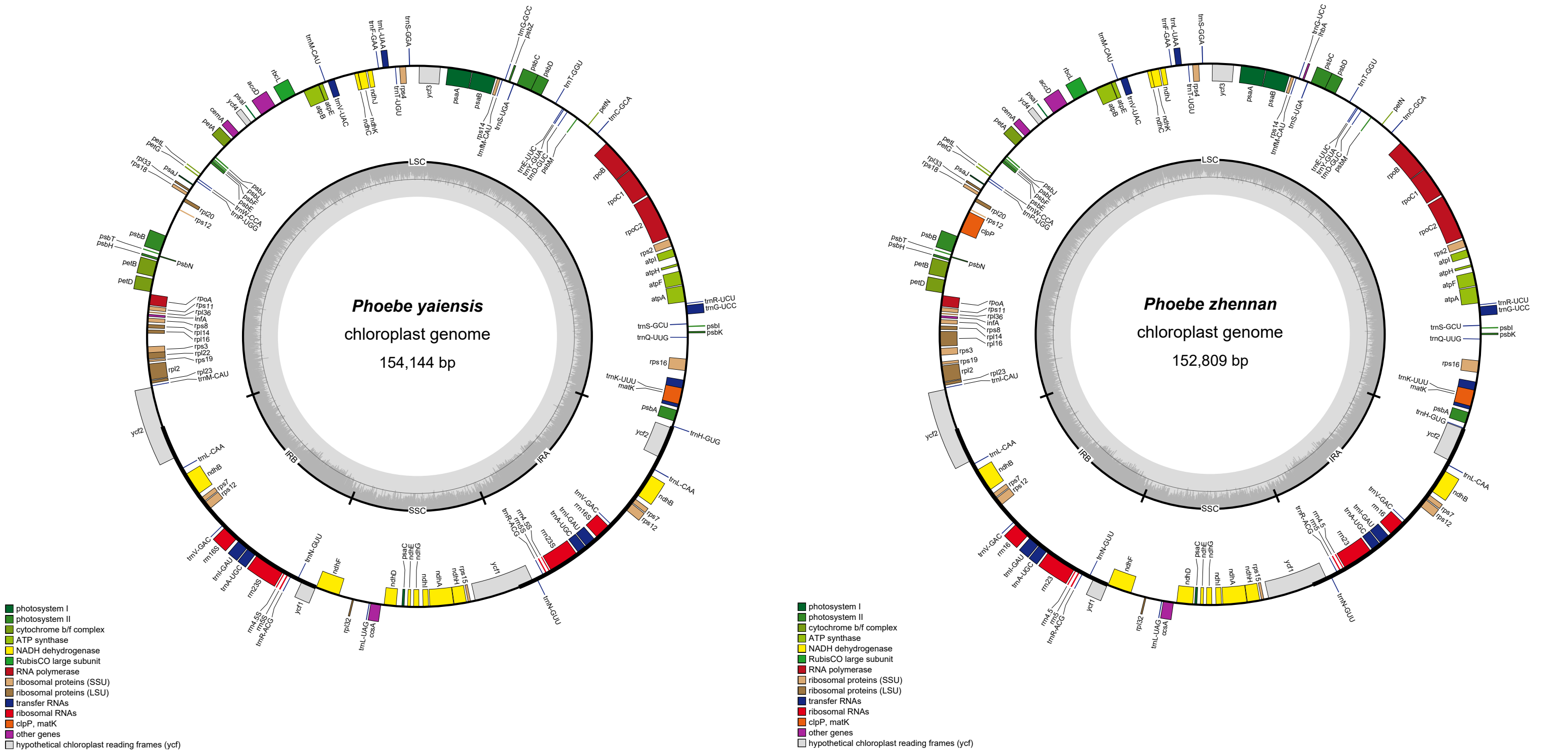


**Figure S1.** Gene maps of plastomes of 20 species of *Phoebe*. The quadripartite structure was shown in large single copy (LSC), small single copy (SSC), and two inverted repeat (IRA and IRB) regions. Genes were shown in different colors at the outer ring according to functional groups, with clockwise-transcribed genes outsides and counterclockwise-transcribed genes insides. GC content were shown in darker gray shade while AT content were in lighter at the inner ring.

(a)

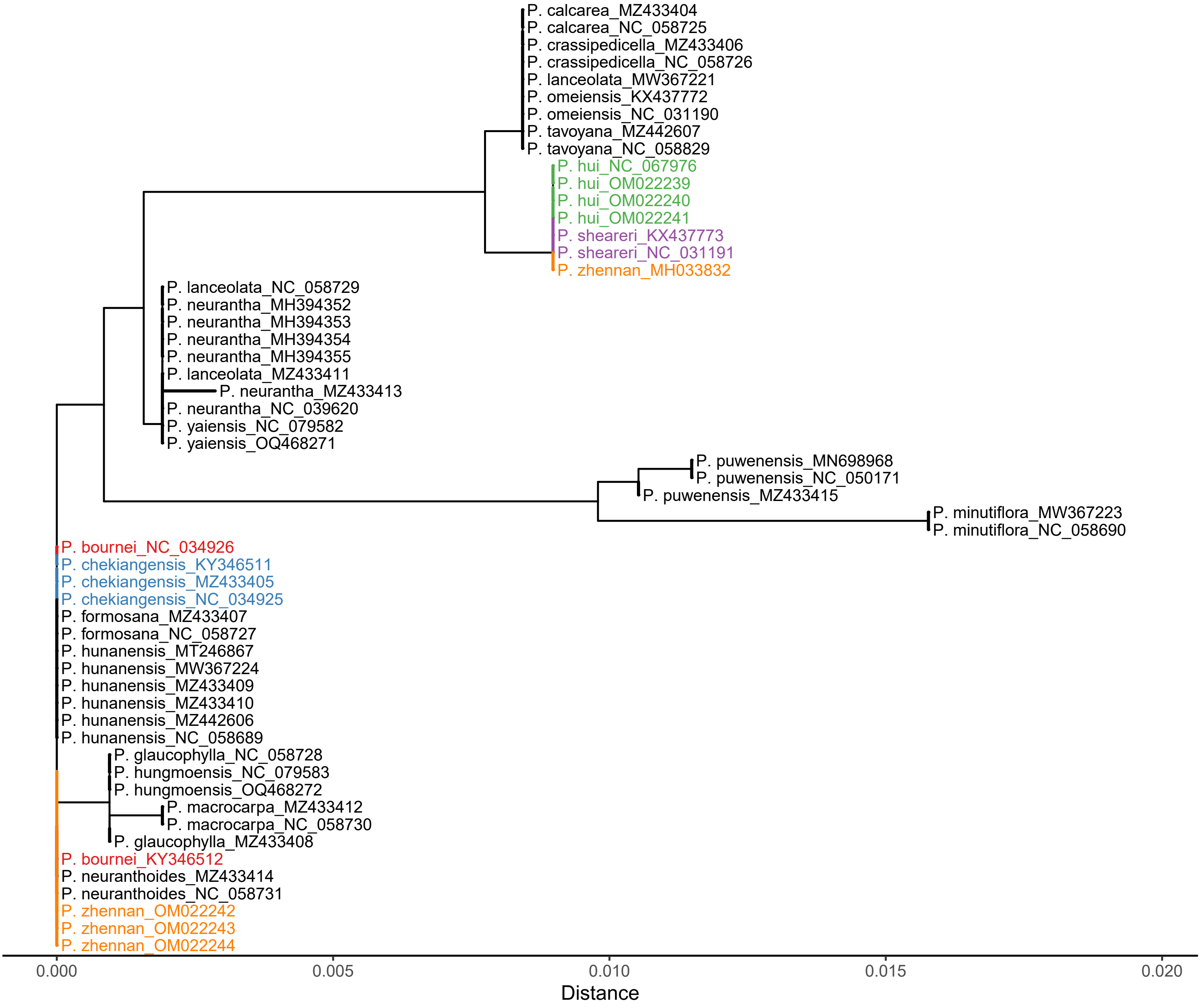

P. calcarea_MZ433404
P. calcarea_NC_058725
P. crassipedicella_MZ433406
P. crassipedicella_NC_058726
P. lanceolata_MW367221
P. omeiensis_KX437772
P. omeiensis_NC_031190
P. tavoyana_MZ442607
P. tavoyana_NC_058829
P. hui_NC_067976
P. hui_OM022239
P. hui_OM022240
P. hui_OM022241
P. sheareri_KX437773
P. sheareri_NC_031191
P. zhennan_MH033832
P. lanceolata_NC_058729
P. neurantha_MH394352
P. neurantha_MH394353
P. neurantha_MH394354
P. neurantha_MH394355
P. lanceolata_MZ433411
P. neurantha_MZ433413
P. neurantha_NC_039620
P. yaiensis_NC_079582
P. yaiensis_OQ468271
P. puwenensis_MN698968
P. puwenensis_NC_050171
P. puwenensis_MZ433415
P. minutiflora_MW367223
P. minutiflora_NC_058690
P. bournei_NC_034926
P. chekiangensis_KY346511
P. chekiangensis_MZ433405
P. chekiangensis_NC_034925
P. formosana_MZ433407
P. formosana_NC_058727
P. hunanensis_MT246867
P. hunanensis_MW367224
P. hunanensis_MZ433409
P. hunanensis_MZ433410
P. hunanensis_MZ442606
P. hunanensis_NC_058689
P. glaucophylla_NC_058728
P. hungmoensis_NC_079583
P. hungmoensis_OQ468272
P. macrocarpa_MZ433412
P. macrocarpa_NC_058730
P. glaucophylla_MZ433408
P. bournei_KY346512
P. neuranthoides_MZ433414
P. neuranthoides_NC_058731
P. zhennan_OM022242
P. zhennan_OM022243
P. zhennan_OM022244
0.000
0.005
0.010
0.015
0.020
Distance

(b)

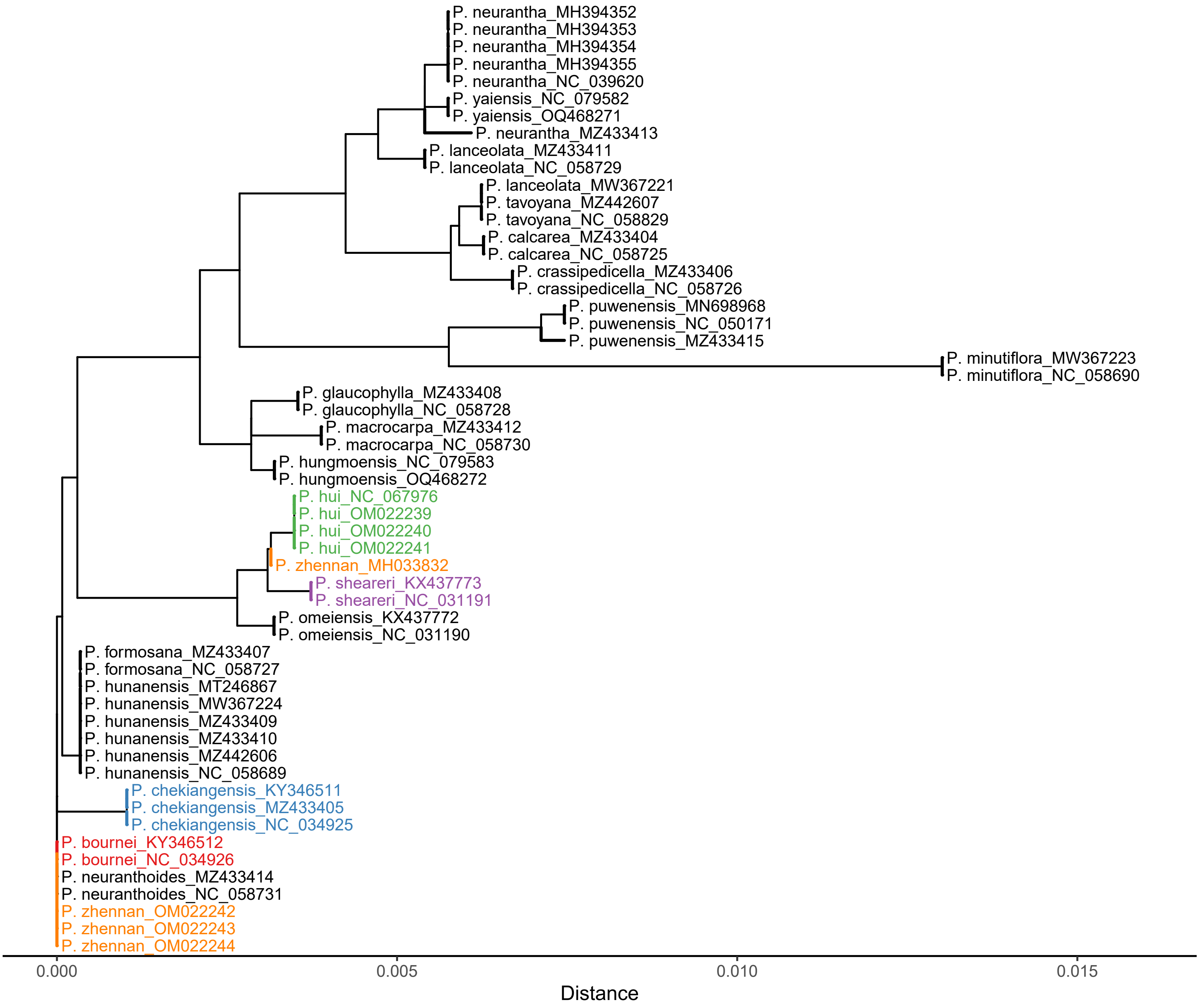
P. neurantha_MH394352
P. neurantha_MH394353
P. neurantha_MH394354
P. neurantha_MH394355
P. neurantha_NC_039620
P. yaiensis_NC_079582
P. yaiensis_OQ468271
P. neurantha_MZ433413
P. lanceolata_MZ433411
P. lanceolata_NC_058729
P. lanceolata_MW367221
P. tavoyana_MZ442607
P. tavoyana_NC_058829
P. calcarea_MZ433404
P. calcarea_NC_058725
P. crassipedicella_MZ433406
P. crassipedicella_NC_058726
P. puwenensis_MN698968
P. puwenensis_NC_050171
P. puwenensis_MZ433415
P. minutiflora_MW367223
P. minutiflora_NC_058690
P. glaucophylla_MZ433408
P. glaucophylla_NC_058728
P. macrocarpa_MZ433412
P. macrocarpa_NC_058730
P. hungmoensis_NC_079583
P. hungmoensis_OQ468272
P. hui_NC_067976
P. hui_OM022239
P. hui_OM022240
P. hui_OM022241
P. zhennan_MH033832
P. sheareri_KX437773
P. sheareri_NC_031191
P. omeiensis_KX437772
P. omeiensis_NC_031190
P. formosana_MZ433407
P. formosana_NC_058727
P. hunanensis_MT246867
P. hunanensis_MW367224
P. hunanensis_MZ433409
P. hunanensis_MZ433410
P. hunanensis_MZ442606
P. hunanensis_NC_058689
P. chekiangensis_KY346511
P. chekiangensis_MZ433405
P. chekiangensis_NC_034925
P. bournei_KY346512
P. bournei_NC_034926
P. neuranthoides_MZ433414
P. neuranthoides_NC_058731
P. zhennan_OM022242
P. zhennan_OM022243
P. zhennan_OM022244
0.000
0.005
0.010
0.015
Distance

(c)

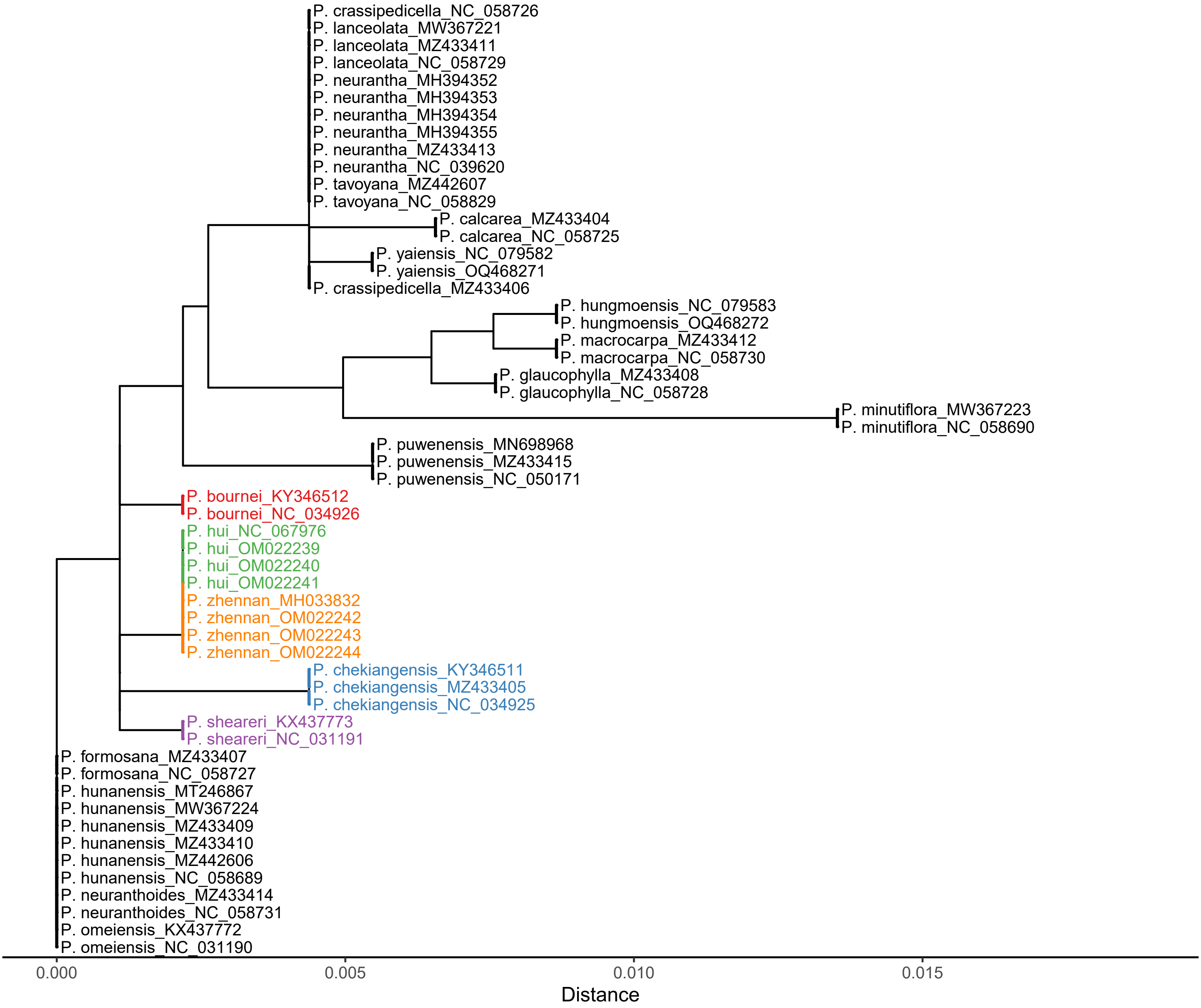
P. crassipedicella_NC_058726
P. lanceolata_MW367221
P. lanceolata_MZ433411
P. lanceolata_NC_058729
P. neurantha_MH394352
P. neurantha_MH394353
P. neurantha_MH394354
P. neurantha_MH394355
P. neurantha_MZ433413
P. neurantha_NC_039620
P. tavoyana_MZ442607
P. tavoyana_NC_058829
P. calcarea_MZ433404
P. calcarea_NC_058725
P. yaiensis_NC_079582
P. yaiensis_OQ468271
P. crassipedicella_MZ433406
P. hungmoensis_NC_079583
P. hungmoensis_OQ468272
P. macrocarpa_MZ433412
P. macrocarpa_NC_058730
P. glaucophylla_MZ433408
P. glaucophylla_NC_058728
P. minutiflora_MW367223
P. minutiflora_NC_058690
P. puwenensis_MN698968
P. puwenensis_MZ433415
P. puwenensis_NC_050171
P. bournei_KY346512
P. bournei_NC_034926
P. hui_NC_067976
P. hui_OM022239
P. hui_OM022240
P. hui_OM022241
P. zhennan_MH033832
P. zhennan_OM022242
P. zhennan_OM022243
P. zhennan_OM022244
P. chekiangensis_KY346511
P. chekiangensis_MZ433405
P. chekiangensis_NC_034925
P. sheareri_KX437773
P. sheareri_NC_031191
P. formosana_MZ433407
P. formosana_NC_058727
P. hunanensis_MT246867
P. hunanensis_MW367224
P. hunanensis_MZ433409
P. hunanensis_MZ433410
P. hunanensis_MZ442606
P. hunanensis_NC_058689
P. neuranthoides_MZ433414
P. neuranthoides_NC_058731
P. omeiensis_KX437772
P. omeiensis_NC_031190
0.000
0.005
0.010
0.015
Distance

(d)

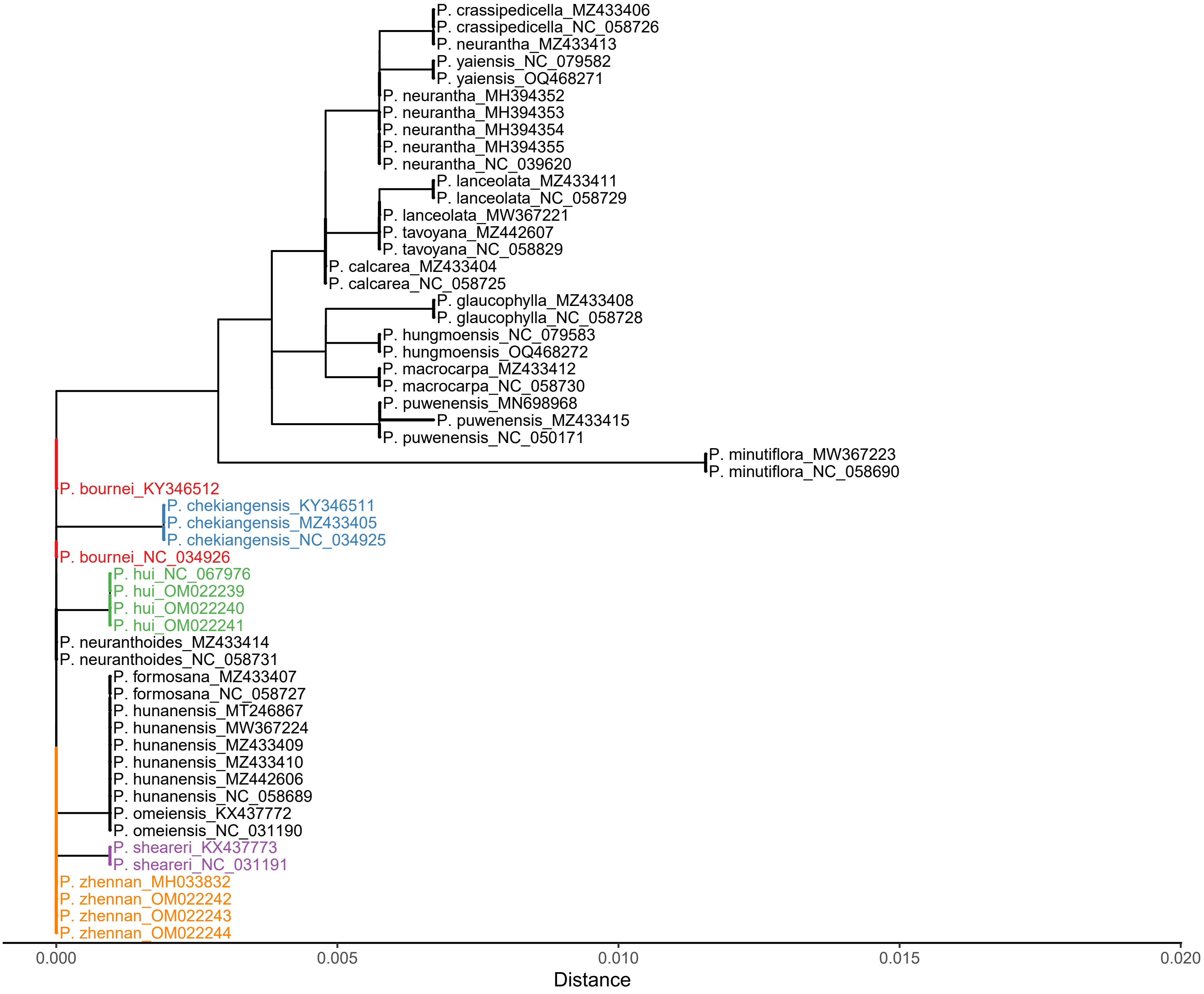


**Figure S2.** Neighbor-joining (NJ) trees based on single locus: (a) *petA-psbJ-psbL-psbF-psbE* (b) Ψ*ycf1-ndhF* (c) *rpl32-trnL*$^{UAG}$ (d) *ycf1*

(a)

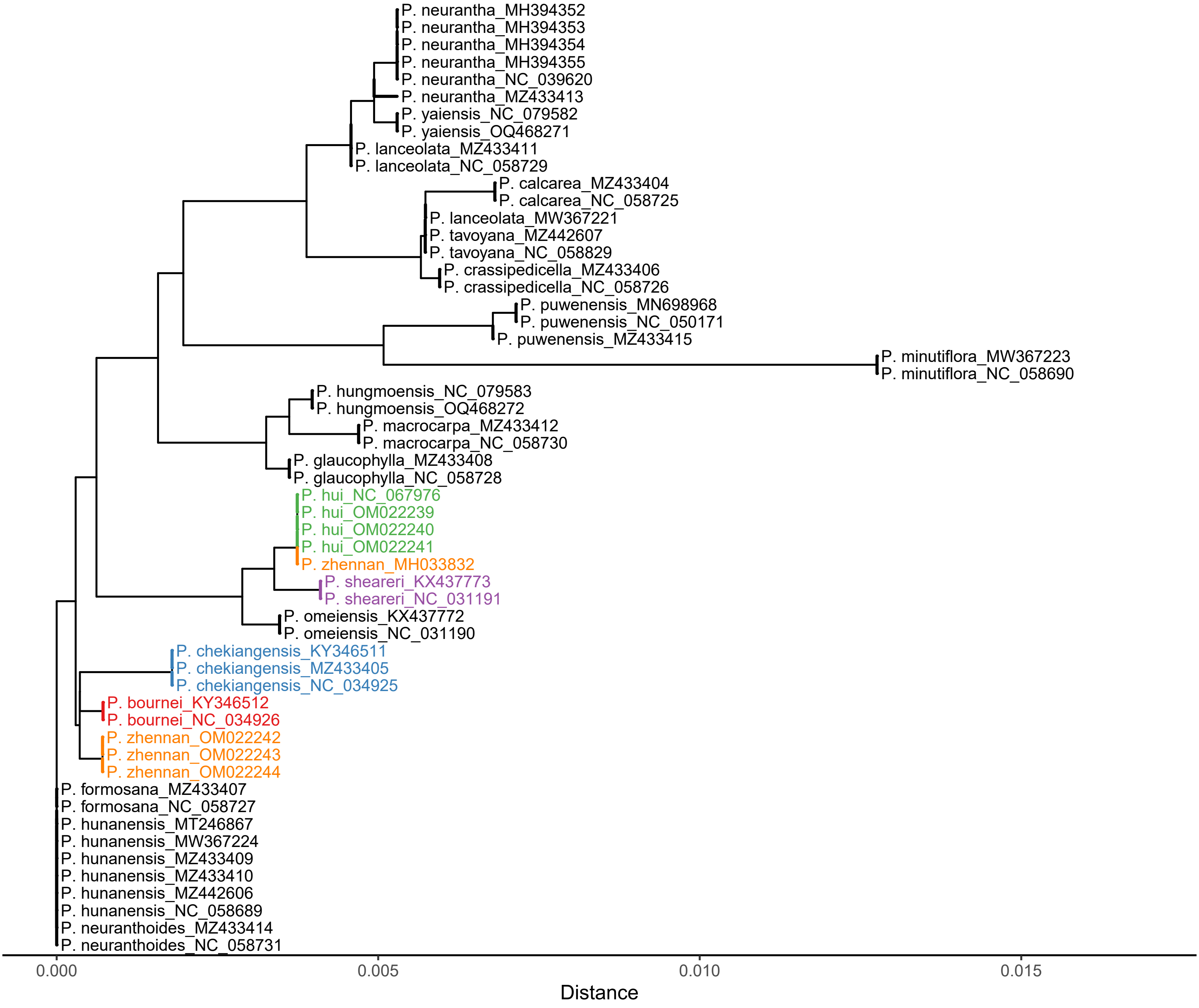
P. neurantha_MH394352
P. neurantha_MH394353
P. neurantha_MH394354
P. neurantha_MH394355
P. neurantha_NC_039620
P. neurantha_MZ433413
P. yaiensis_NC_079582
P. yaiensis_OQ468271
P. lanceolata_MZ433411
P. lanceolata_NC_058729
P. calcarea_MZ433404
P. calcarea_NC_058725
P. lanceolata_MW367221
P. tavoyana_MZ442607
P. tavoyana_NC_058829
P. crassipedicella_MZ433406
P. crassipedicella_NC_058726
P. puwenensis_MN698968
P. puwenensis_NC_050171
P. puwenensis_MZ433415
P. minutiflora_MW367223
P. minutiflora_NC_058690
P. hungmoensis_NC_079583
P. hungmoensis_OQ468272
P. macrocarpa_MZ433412
P. macrocarpa_NC_058730
P. glaucophylla_MZ433408
P. glaucophylla_NC_058728
P. hui_NC_067976
P. hui_OM022239
P. hui_OM022240
P. hui_OM022241
P. zhennan_MH033832
P. sheareri_KX437773
P. sheareri_NC_031191
P. omeiensis_KX437772
P. omeiensis_NC_031190
P. chekiangensis_KY346511
P. chekiangensis_MZ433405
P. chekiangensis_NC_034925
P. bournei_KY346512
P. bournei_NC_034926
P. zhennan_OM022242
P. zhennan_OM022243
P. zhennan_OM022244
P. formosana_MZ433407
P. formosana_NC_058727
P. hunanensis_MT246867
P. hunanensis_MW367224
P. hunanensis_MZ433409
P. hunanensis_MZ433410
P. hunanensis_MZ442606
P. hunanensis_NC_058689
P. neuranthoides_MZ433414
P. neuranthoides_NC_058731
0.000
0.005
0.010
0.015
Distance

(b)

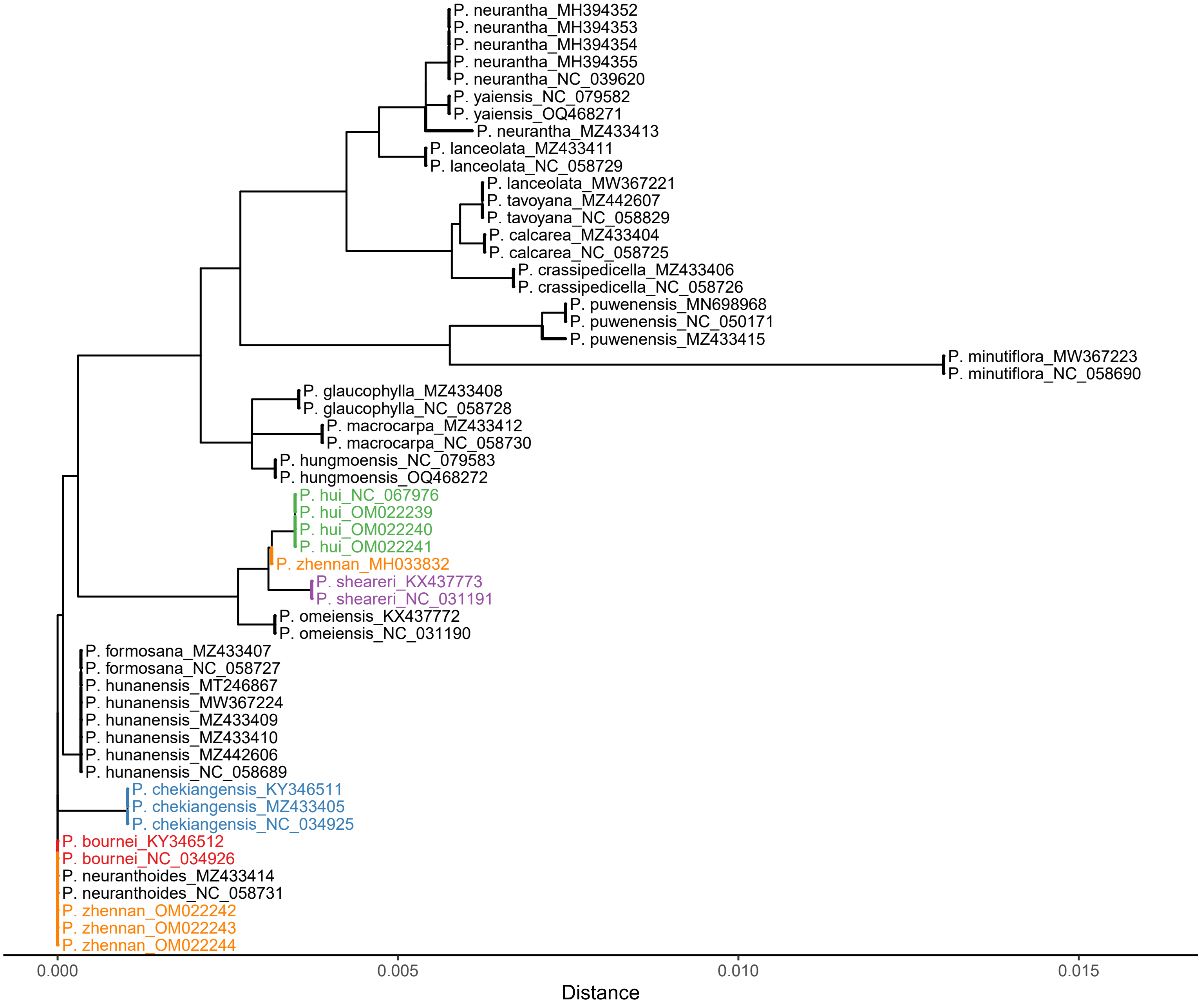

P. neurantha_MH394352
P. neurantha_MH394353
P. neurantha_MH394354
P. neurantha_MH394355
P. neurantha_NC_039620
P. yaiensis_NC_079582
P. yaiensis_OQ468271
P. neurantha_MZ433413
P. lanceolata_MZ433411
P. lanceolata_NC_058729
P. lanceolata_MW367221
P. tavoyana_MZ442607
P. tavoyana_NC_058829
P. calcarea_MZ433404
P. calcarea_NC_058725
P. crassipedicella_MZ433406
P. crassipedicella_NC_058726
P. puwenensis_MN698968
P. puwenensis_NC_050171
P. puwenensis_MZ433415
P. minutiflora_MW367223
P. minutiflora_NC_058690
P. glaucophylla_MZ433408
P. glaucophylla_NC_058728
P. macrocarpa_MZ433412
P. macrocarpa_NC_058730
P. hungmoensis_NC_079583
P. hungmoensis_OQ468272
P. hui_NC_067976
P. hui_OM022239
P. hui_OM022240
P. hui_OM022241
P. zhennan_MH033832
P. sheareri_KX437773
P. sheareri_NC_031191
P. omeiensis_KX437772
P. omeiensis_NC_031190
P. formosana_MZ433407
P. formosana_NC_058727
P. hunanensis_MT246867
P. hunanensis_MW367224
P. hunanensis_MZ433409
P. hunanensis_MZ433410
P. hunanensis_MZ442606
P. hunanensis_NC_058689
P. chekiangensis_KY346511
P. chekiangensis_MZ433405
P. chekiangensis_NC_034925
P. bournei_KY346512
P. bournei_NC_034926
P. neuranthoides_MZ433414
P. neuranthoides_NC_058731
P. zhennan_OM022242
P. zhennan_OM022243
P. zhennan_OM022244
0.000
0.005
0.010
0.015
Distance

(c)

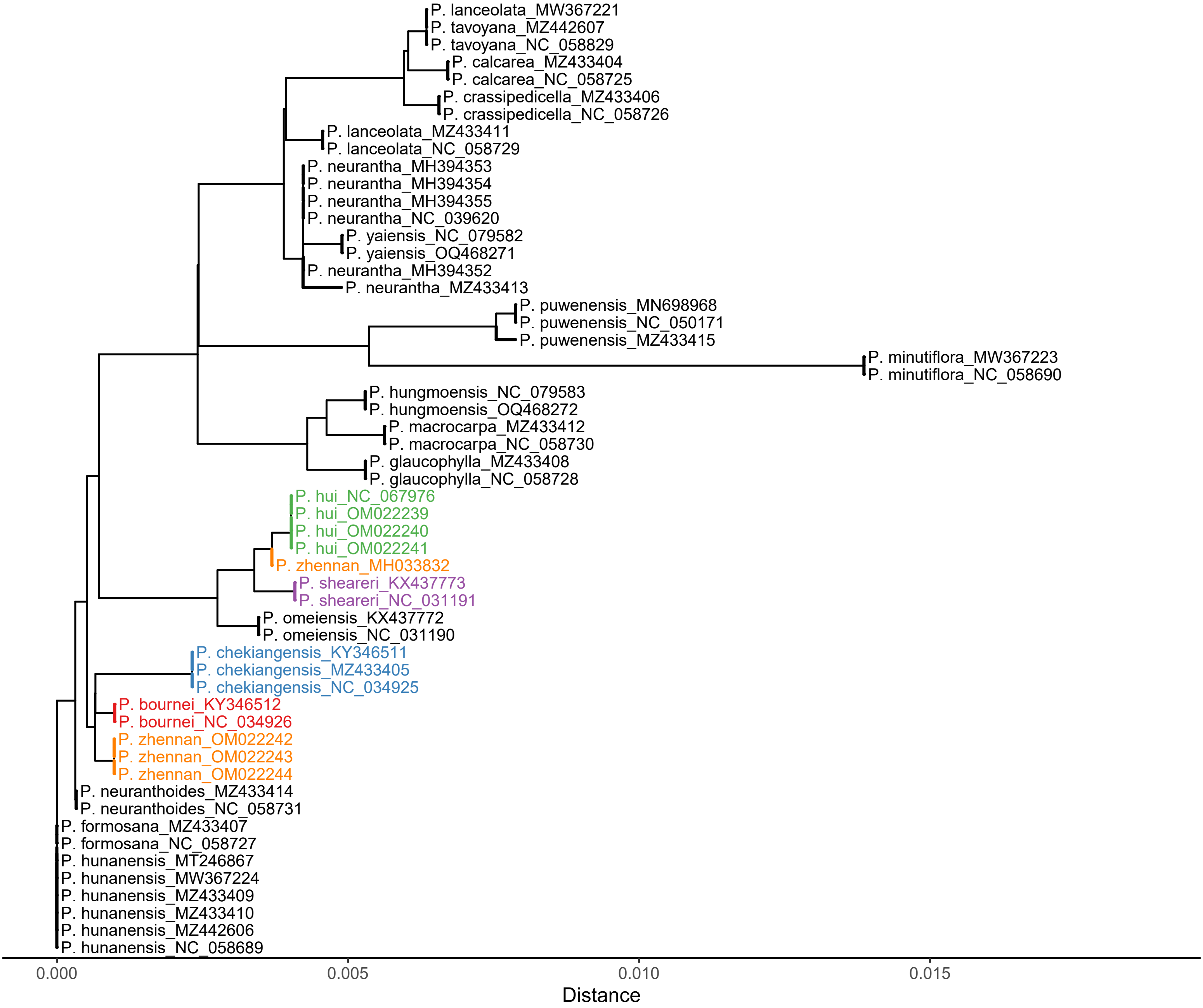
P. lanceolata_MW367221
P. tavoyana_MZ442607
P. tavoyana_NC_058829
P. calcarea_MZ433404
P. calcarea_NC_058725
P. crassipedicella_MZ433406
P. crassipedicella_NC_058726
P. lanceolata_MZ433411
P. lanceolata_NC_058729
P. neurantha_MH394353
P. neurantha_MH394354
P. neurantha_MH394355
P. neurantha_NC_039620
P. yaiensis_NC_079582
P. yaiensis_OQ468271
P. neurantha_MH394352
P. neurantha_MZ433413
P. puwenensis_MN698968
P. puwenensis_NC_050171
P. puwenensis_MZ433415
P. minutiflora_MW367223
P. minutiflora_NC_058690
P. hungmoensis_NC_079583
P. hungmoensis_OQ468272
P. macrocarpa_MZ433412
P. macrocarpa_NC_058730
P. glaucophylla_MZ433408
P. glaucophylla_NC_058728
P. hui_NC_067976
P. hui_OM022239
P. hui_OM022240
P. hui_OM022241
P. zhennan_MH033832
P. sheareri_KX437773
P. sheareri_NC_031191
P. omeiensis_KX437772
P. omeiensis_NC_031190
P. chekiangensis_KY346511
P. chekiangensis_MZ433405
P. chekiangensis_NC_034925
P. bournei_KY346512
P. bournei_NC_034926
P. zhennan_OM022242
P. zhennan_OM022243
P. zhennan_OM022244
P. neuranthoides_MZ433414
P. neuranthoides_NC_058731
P. formosana_MZ433407
P. formosana_NC_058727
P. hunanensis_MT246867
P. hunanensis_MW367224
P. hunanensis_MZ433409
P. hunanensis_MZ433410
P. hunanensis_MZ442606
P. hunanensis_NC_058689
0.000
0.005
0.010
0.015
Distance

(d)

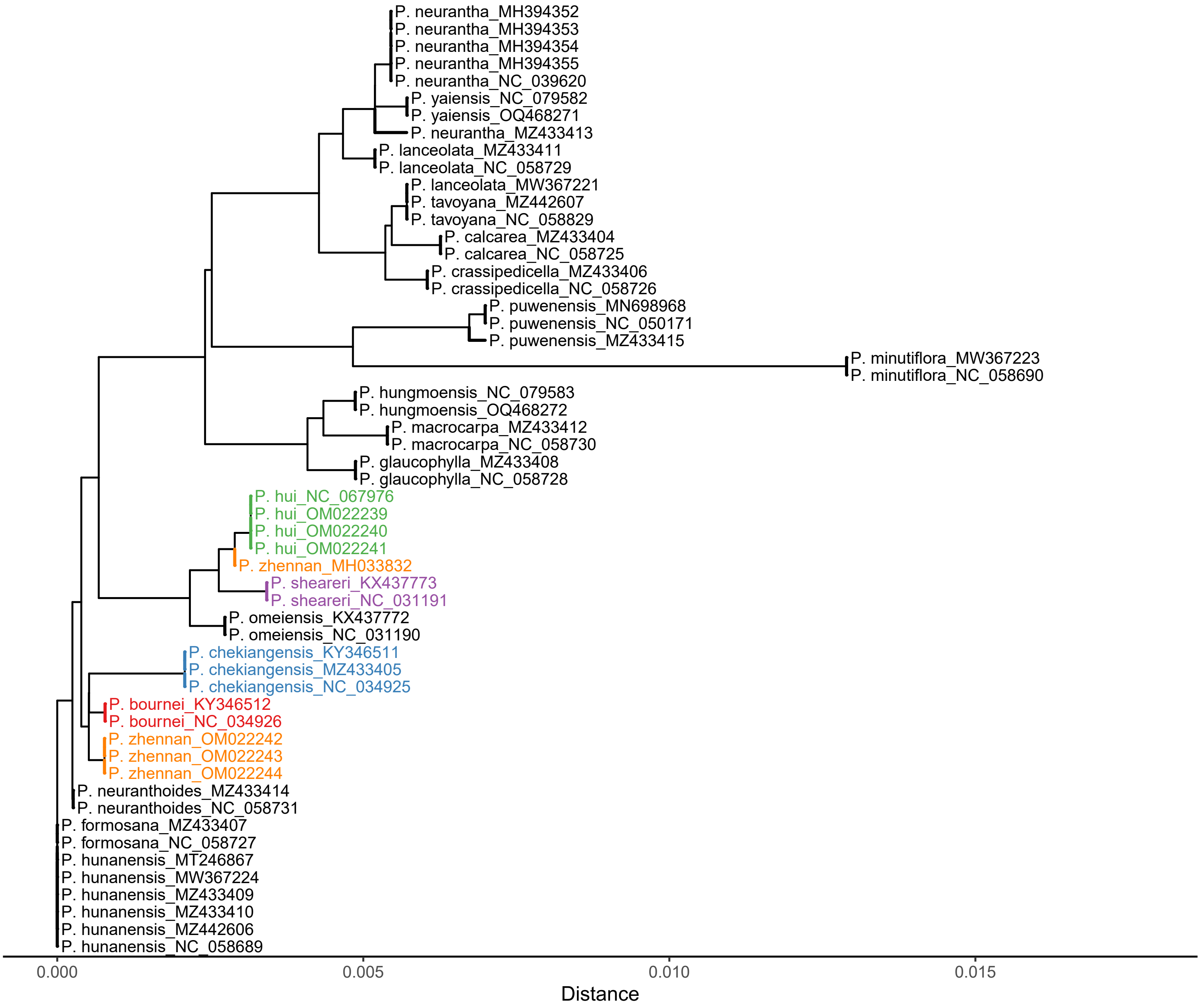


**Figure S3.** Neighbor-joining (NJ) trees based on multi loci: (a) P+F+R (b) P+F+Y (c) P +R+Y (d) P+F+R+Y. P: *petA-psbJ-psbL-psbF-psbE*; F: Ψ*ycf1-ndhF*; R:*rpl32-trnL*$^{UAG}$; Y:*ycf1*.

**Table S1** Summary of *Phoebe species* included in two lists

| Species | China's National Key Protected Wild Plant List[1] | IUCN[2] Red List of Threatened Species[3] |
|---|---|---|
| *P. bournei* | Category II | -Not included- |
| *P. chekiangensis* | Category II | Vulnerable（VU） |
| *P. hui* | Category II | -Not included- |
| *P. sheareri* | -Not included- | Least Concern |
| *P. zhennan* | Category II | Vulnerable（VU） |

[1] https://www.forestry.gov.cn/c/www/gkml/11057.jhtml

[2] IUCN:The International Union for Conservation of Nature's

[3] https://www.iucnredlist.org/

**Table S2** Characteristic of chloroplast genomes of *Phoebe*

| species | accession number | GC content | count | | | | length (bp) | | | | |
|---|---|---|---|---|---|---|---|---|---|---|---|
| | | | gene | CDS | rRNA | tRNA | total | LSC | SSC | IRA | IRB |
| *P. bournei* | KY346512 | 39.10% | 127 | 81 | 8 | 36 | 152,853 | 93,777 | 18,928 | 20,074 | 20,074 |
| *P. bournei* | NC_034926 | 39.10% | 127 | 81 | 8 | 36 | 152,853 | 93,777 | 18,928 | 20,074 | 20,074 |
| *P. calcarea* | MZ433404 | 39.20% | 126 | 82 | 8 | 36 | 152,851 | 93,761 | 18,904 | 20,093 | 20,093 |
| *P. calcarea* | NC_058725 | 39.20% | 126 | 82 | 8 | 36 | 152,851 | 93,761 | 18,904 | 20,093 | 20,093 |
| *P. chekiangensis* | KY346511 | 39.10% | 127 | 81 | 8 | 36 | 152,849 | 93,772 | 18,895 | 20,091 | 20,091 |
| *P. chekiangensis* | MZ433405 | 39.10% | 126 | 81 | 8 | 36 | 152,870 | 93,750 | 18,910 | 20,105 | 20,105 |
| *P. chekiangensis* | NC_034925 | 39.10% | 127 | 81 | 8 | 36 | 152,849 | 93,772 | 18,895 | 20,091 | 20,091 |
| *P. crassipedicella* | MZ433406 | 39.20% | 126 | 81 | 8 | 36 | 152,784 | 93,729 | 18,905 | 20,075 | 20,075 |
| *P. crassipedicella* | NC_058726 | 39.20% | 126 | 81 | 8 | 36 | 152,784 | 93,729 | 18,905 | 20,075 | 20,075 |
| *P. formosana* | MZ433407 | 39.10% | 126 | 82 | 8 | 36 | 152,698 | 95,694 | 18,830 | 19,087 | 19,087 |
| *P. formosana* | NC_058727 | 39.10% | 126 | 82 | 8 | 36 | 152,698 | 95,694 | 18,830 | 19,087 | 19,087 |
| *P. glaucophylla* | MZ433408 | 39.10% | 126 | 82 | 8 | 36 | 152,859 | 93,750 | 18,923 | 20,093 | 20,093 |
| *P. glaucophylla* | NC_058728 | 39.10% | 126 | 82 | 8 | 36 | 152,859 | 93,750 | 18,919 | 20,095 | 20,095 |
| *P. hui* | NC_067976 | 39.10% | 127 | 81 | 8 | 36 | 152,837 | 93,751 | 18,936 | 20,075 | 20,075 |
| *P. hui* | OM022239 | 39.10% | 127 | 81 | 8 | 36 | 152,837 | 93,751 | 18,936 | 20,075 | 20,075 |
| *P. hui* | OM022240 | 39.10% | 127 | 81 | 8 | 36 | 152,831 | 93,751 | 18,930 | 20,075 | 20,075 |
| *P. hui* | OM022241 | 39.10% | 127 | 81 | 8 | 36 | 152,831 | 93,751 | 18,930 | 20,075 | 20,075 |
| *P. hunanensis* | MT246867 | 39.10% | 127 | 82 | 8 | 36 | 152,791 | 93,713 | 18,928 | 20,075 | 20,075 |
| *P. hunanensis* | MW367224 | 39.20% | 127 | 81 | 8 | 36 | 154,147 | 93,713 | 18,928 | 20,753 | 20,753 |
| *P. hunanensis* | MZ433409 | 39.10% | 126 | 82 | 8 | 36 | 152,771 | 93,713 | 18,928 | 20,065 | 20,065 |
| *P. hunanensis* | MZ433410 | 39.10% | 126 | 82 | 8 | 36 | 152,798 | 93,720 | 18,928 | 20,075 | 20,075 |
| *P. hunanensis* | MZ442606 | 39.10% | 128 | 84 | 8 | 36 | 152,791 | 93,713 | 18,928 | 20,075 | 20,075 |
| *P. hunanensis* | NC_058689 | 39.10% | 127 | 82 | 8 | 36 | 152,791 | 93,713 | 18,928 | 20,075 | 20,075 |
| *P. hungmoensis* | NC_079583 | 39.10% | 125 | 81 | 8 | 36 | 152,785 | 93,708 | 18,873 | 20,102 | 20,102 |
| *P. hungmoensis* | OQ468272 | 39.10% | 125 | 81 | 8 | 36 | 152,785 | 93,708 | 18,873 | 20,102 | 20,102 |
| *P. lanceolata* | MW367221 | 39.20% | 127 | 81 | 8 | 36 | 154,169 | 93,769 | 18,898 | 20,751 | 20,751 |
| *P. lanceolata* | MZ433411 | 39.20% | 126 | 82 | 8 | 36 | 152,809 | 93,765 | 18,898 | 20,073 | 20,073 |
| *P. lanceolata* | NC_058729 | 39.20% | 126 | 82 | 8 | 36 | 152,809 | 93,765 | 18,898 | 20,073 | 20,073 |
| *P. macrocarpa* | MZ433412 | 39.10% | 126 | 82 | 8 | 36 | 152,750 | 93,725 | 18,839 | 20,093 | 20,093 |
| *P. macrocarpa* | NC_058730 | 39.10% | 126 | 82 | 8 | 36 | 152,750 | 93,725 | 18,839 | 20,093 | 20,093 |
| *P. minutiflora* | MW367223 | 39.20% | 127 | 81 | 8 | 36 | 152,654 | 93,697 | 18,809 | 20,074 | 20,074 |
| *P. minutiflora* | NC_058690 | 39.20% | 127 | 81 | 8 | 36 | 152,654 | 93,697 | 18,809 | 20,074 | 20,074 |
| *P. neurantha* | MH394352 | 39.20% | 126 | 82 | 6 | 36 | 152,782 | 93,730 | 18,904 | 20,074 | 20,074 |
| *P. neurantha* | MH394353 | 39.20% | 126 | 82 | 6 | 36 | 152,782 | 93,730 | 18,904 | 20,074 | 20,074 |
| *P. neurantha* | MH394354 | 39.10% | 126 | 82 | 6 | 36 | 152,782 | 93,730 | 18,904 | 20,074 | 20,074 |
| *P. neurantha* | MH394355 | 39.20% | 126 | 82 | 6 | 36 | 152,782 | 93,730 | 18,904 | 20,074 | 20,074 |
| *P. neurantha* | MZ433413 | 39.20% | 126 | 82 | 8 | 36 | 152,815 | 93,730 | 18,904 | 20,091 | 20,091 |
| *P. neurantha* | NC_039620 | 39.20% | 126 | 82 | 6 | 36 | 152,782 | 93,730 | 18,902 | 20,075 | 20,075 |
| *P. neuranthoides* | MZ433414 | 39.10% | 126 | 82 | 8 | 36 | 152,880 | 93,781 | 18,949 | 20,075 | 20,075 |
| *P. neuranthoides* | NC_058731 | 39.10% | 126 | 82 | 8 | 36 | 152,880 | 93,781 | 18,949 | 20,075 | 20,075 |
| *P. omeiensis* | KX437772 | 39.10% | 127 | 81 | 8 | 36 | 152,855 | 93,966 | 18,929 | 19,980 | 19,980 |
| *P. omeiensis* | NC_031190 | 39.10% | 127 | 81 | 8 | 36 | 152,855 | 93,966 | 18,929 | 19,980 | 19,980 |

**Table S2** (Continued)

| species | accession number | GC content | count | | | | length (bp) | | | | |
|---|---|---|---|---|---|---|---|---|---|---|---|
| | | | gene | CDS | rRNA | tRNA | total | LSC | SSC | IRA | IRB |
| *P. puwenensis* | MN698968 | 39.10% | 127 | 82 | 8 | 36 | 152,746 | 93,685 | 18,909 | 20,076 | 20,076 |
| *P. puwenensis* | MZ433415 | 39.10% | 126 | 82 | 8 | 36 | 152,746 | 93,685 | 18,909 | 20,076 | 20,076 |
| *P. puwenensis* | NC_050171 | 39.10% | 127 | 82 | 8 | 36 | 152,746 | 93,685 | 18,909 | 20,076 | 20,076 |
| *P. sheareri* | KX437773 | 39.10% | 127 | 81 | 8 | 36 | 152,876 | 93,893 | 18,915 | 20,034 | 20,034 |
| *P. sheareri* | NC_031191 | 39.10% | 127 | 81 | 8 | 36 | 152,876 | 93,893 | 18,915 | 20,034 | 20,034 |
| *P. tavoyana* | MZ442607 | 39.20% | 128 | 84 | 8 | 36 | 152,814 | 93,769 | 18,899 | 20,073 | 20,073 |
| *P. tavoyana* | NC_058829 | 39.20% | 128 | 84 | 8 | 36 | 152,814 | 93,769 | 18,899 | 20,073 | 20,073 |
| *P. yaiensis* | NC_079582 | 39.20% | 127 | 83 | 8 | 36 | 154,144 | 93,732 | 18,906 | 20,753 | 20,753 |
| *P. yaiensis* | OQ468271 | 39.20% | 127 | 83 | 8 | 36 | 154,144 | 93,732 | 18,906 | 20,753 | 20,753 |
| *P. zhennan* | MH033832 | 39.10% | 127 | 81 | 8 | 36 | 152,809 | 93,729 | 18,930 | 20,075 | 20,075 |
| *P. zhennan* | OM022242 | 39.10% | 127 | 81 | 8 | 36 | 152,830 | 93,752 | 18,928 | 20,075 | 20,075 |
| *P. zhennan* | OM022243 | 39.10% | 127 | 81 | 8 | 36 | 152,830 | 93,752 | 18,928 | 20,075 | 20,075 |
| *P. zhennan* | OM022244 | 39.10% | 127 | 81 | 8 | 36 | 152,830 | 93,752 | 18,928 | 20,075 | 20,075 |

**Table S3** The 33 samples used to test primer*(rpl32-trnL*$^{UAG}$, *ycf1*) universality

| NO.. | Material | Voucher | PCR success(R) | | PCR success(Y) | | leaf (R&Y) |
|---|---|---|---|---|---|---|---|
| | | | Yes | No | Yes | No | |
| 1 | | PB4 | Yes | | Yes | | Yes |
| 2 | *P. bournei* (PB) | PB5 | Yes | | Yes | | |
| 3 | | PB15 | | No | Yes | | |
| 4 | | PB18 | Yes | | Yes | | |
| 5 | | PC4 | Yes | | Yes | | |
| 6 | | PC5 | Yes | | Yes | | |
| 7 | | PC6 | Yes | | Yes | | |
| 8 | | PC7 | Yes | | Yes | | |
| 9 | *P. chekiangensis* (PC) | PC8 | Yes | | Yes | | |
| 10 | | PC9 | Yes | | Yes | | |
| 11 | | PC10 | Yes | | Yes | | Yes |
| 12 | | PC11 | Yes | | Yes | | |
| 13 | | PC12 | Yes | | Yes | | |
| 14 | | PH5 | Yes | | Yes | | |
| 15 | *P. hui* (PH) | PH6 | Yes | | Yes | | |
| 16 | | PH7 | | No | Yes | | |
| 17 | | PH3 | Yes | | Yes | | Yes |
| 18 | | PS1 | Yes | | Yes | | |
| 19 | | PS2 | Yes | | Yes | | |
| 20 | | PS3 | Yes | | Yes | | |
| 21 | | PS4 | Yes | | Yes | | Yes |
| 22 | *P. sheareri* (PS) | PS5 | Yes | | Yes | | |
| 23 | | PS8 | Yes | | Yes | | |
| 24 | | PS9 | Yes | | Yes | | |
| 25 | | PS10 | Yes | | Yes | | |
| 26 | | PS11 | Yes | | Yes | | |
| 27 | | PZ1 | Yes | | Yes | | Yes |
| 28 | | PZ2 | Yes | | Yes | | |
| 29 | | PZ3 | Yes | | Yes | | |
| 30 | *P. zhennan* (PZ) | PZ12 | Yes | | Yes | | |
| 31 | | PZ13 | | No | | No | |
| 32 | | PZ14 | | No | | No | |
| 33 | | PZ15 | Yes | | Yes | | |
| The amplification rates | | | 87.88% | | 93.94% | | |

R: *rpl32-trnL*$^{UAG}$; Y: *ycf1*

**Table S4** Samples source information

| Material | Voucher | Location |
|---|---|---|
| *P. bournei* (PB) | PB4 (leaf *) \PB5 | Zhejiang Agriculture and Forestry University, Hangzhou, Zhejiang Province (30.26N, 119.73E) |
| | PB15 | Jindong Forest Farm, Yongzhou, Hunan Province (26.29N, 112.09E) |
| | PB18 | Qingyuan Forest Farm, Lishui , Zhejiang Province (27.61N, 119.06E) |
| *P. chekiangensis* (PC) | PC4\PC5\PC6 | Jiande Shouchang Forest Farm, Hangzhou, Zhejiang Province (29.36N, 119.22E) |
| | PC7\PC8\PC9 | Zhejiang Agriculture and Forestry University, Hangzhou, Zhejiang Province (30.26N, 119.73E) |
| | PC10 (leaf *) \PC11\PC12 | Qingyuan Forest Farm, Lishui , Zhejiang Province (27.61N, 119.06E) |
| *P. hui* (PH) | PH5\PH6\PH7 | Wenjiang District, Chengdu, Sichuan Province (30.69N, 103.83E) |
| | PH3 (leaf *) | Dujiangyan, Chengdu, Sichuan Province (30.99N, 103.61E) |
| *P. sheareri* (PS) | PS1\PS2\PS3 | Jiande Shouchang Forest Farm, Hangzhou, Zhejiang Province (29.36N, 119.22E) |
| | PS4 (leaf *) \PS5 | Zhejiang Agriculture and Forestry University, Hangzhou, Zhejiang Province (30.26N, 119.73E) |
| | PS8\PS9\PS10 | Qingyuan Forest Farm, Lishui , Zhejiang Province (27.61N, 119.06E) |
| | PS11 | Zhejiang Academy of Forestry,Hangzhou, Zhejiang Province (30.26N, 119.73E) |
| *P. zhennan* (PZ) | PZ1 \PZ2 (leaf *) \PZ3 | Jiande Shouchang Forest Farm, Hangzhou, Zhejiang Province (29.36N, 119.22E) |
| | PZ12\PZ13\PZ14 | Dujiangyan, Chengdu, Sichuan Province (30.99° N, 103.61° E) |
| | PZ15 | Wenjiang, Chengdu, Sichuan Province (30.69° N, 103.83° E) |

*： wood and leaf samples
