# Supplementary material for: DNA Barcoding Provides Taxonomic Clues for Identifying Five Endangered Phoebe Species in Southern China
Source: Plants (Basel). 2025 Sep 18;14(18):2895. doi: 10.3390/plants14182895 (PMC12473773; doi:10.3390/plants14182895)
Supplement: Supplementary file 1 [file plants-14-02895-s001.zip › Supplementary material 2.pdf]

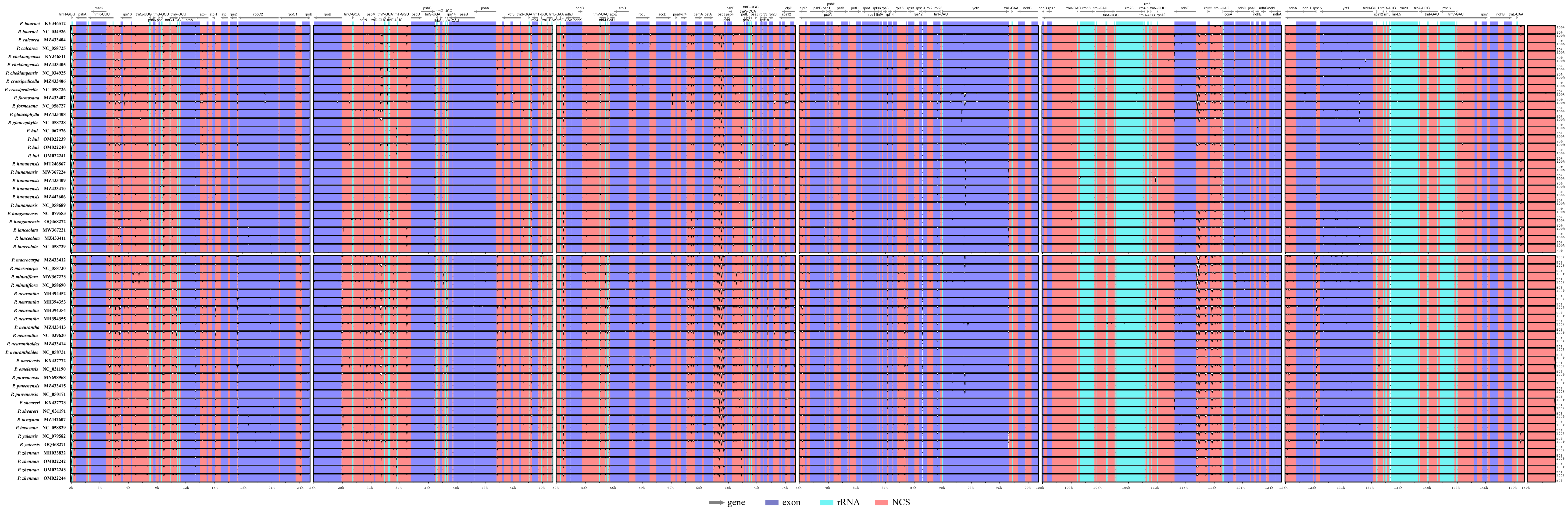

Sequence identity plots among 55 plastomes from 20 *Phoebe* species. The orientation of genes were shown as gray arrows above the alignment. Exon, ribosomal RNA genes (rRNAs) and non-coding sequences (NCSs) were colored in alignment as purple, light-blue and pink blocks respectively. Variation among the chloroplast genomes were highlighted with white peaks.
